# Modelling temporal dynamics of *Culicoides* Latreille (Diptera: Ceratopogonidae) populations on Reunion Island (Indian Ocean), vectors of viruses of veterinary importance

**DOI:** 10.1186/s13071-019-3812-1

**Published:** 2019-11-27

**Authors:** Yannick Grimaud, Hélène Guis, Frédéric Chiroleu, Floriane Boucher, Annelise Tran, Ignace Rakotoarivony, Maxime Duhayon, Catherine Cêtre-Sossah, Olivier Esnault, Eric Cardinale, Claire Garros

**Affiliations:** 1 GDS Réunion, 1 rue du Père Hauck, 97418 La Plaine des Cafres, La Réunion France; 2 University of Reunion Island, 15 avenue René Cassin, 97715 Sainte-Clotilde, La Réunion France; 3 CIRAD, UMR ASTRE, 97490 Sainte-Clotilde, La Réunion France; 4 CIRAD, UMR ASTRE, 101 Antananarivo, Madagascar; 5 ASTRE, University of Montpellier, CIRAD, INRA, Montpellier, France; 6 Epidemiology and clinical research unit, Institut Pasteur of Madagascar, Antananarivo, Madagascar; 7 FOFIFA DRZVP, Antananarivo, Madagascar; 8 CIRAD, UMR PBVMT, 97410 Saint-Pierre, La Réunion France; 9 CIRAD, UMR TETIS, 97490 Sainte-Clotilde, La Réunion France; 10 TETIS, University of Montpellier, Montpellier, France; 11 CIRAD, UMR ASTRE, 34398 Montpellier, France

**Keywords:** *Culicoides*, Temporal dynamics, Hurdle model, Reunion Island, Bluetongue, Epizootic hemorrhagic disease, Indian Ocean

## Abstract

**Background:**

Reunion Island regularly faces outbreaks of epizootic haemorrhagic disease (EHD) and bluetongue (BT), two viral diseases transmitted by haematophagous midges of the genus *Culicoides* (Diptera: Ceratopogonidae) to ruminants. To date, five species of *Culicoides* are recorded in Reunion Island in which the first two are proven vector species: *Culicoides bolitinos*, *C. imicola*, *C. enderleini*, *C. grahamii* and *C. kibatiensis*. Meteorological and environmental factors can severely constrain *Culicoides* populations and activities and thereby affect dispersion and intensity of transmission of *Culicoides*-borne viruses. The aim of this study was to describe and predict the temporal dynamics of all *Culicoides* species present in Reunion Island.

**Methods:**

Between 2016 and 2018, 55 biweekly *Culicoides* catches using Onderstepoort Veterinary Institute traps were set up in 11 sites. A hurdle model (i.e. a presence/absence model combined with an abundance model) was developed for each species in order to determine meteorological and environmental drivers of presence and abundance of *Culicoides*.

**Results:**

Abundance displayed very strong heterogeneity between sites. Average *Culicoides* catch per site per night ranged from 4 to 45,875 individuals. *Culicoides imicola* was dominant at low altitude and *C. kibatiensis* at high altitude. A marked seasonality was observed for the three other species with annual variations. Twelve groups of variables were tested. It was found that presence and/or abundance of all five *Culicoides* species were driven by common parameters: rain, temperature, vegetation index, forested environment and host density. Other parameters such as wind speed and farm building opening size governed abundance level of some species. In addition, *Culicoides* populations were also affected by meteorological parameters and/or vegetation index with different lags of time, suggesting an impact on immature stages. Taking into account all the parameters for the final hurdle model, the error rate by Normalized Root mean Square Error ranged from 4.4 to 8.5%.

**Conclusions:**

To our knowledge, this is the first study to model *Culicoides* population dynamics in Reunion Island. In the absence of vaccination and vector control strategies, determining periods of high abundance of *Culicoides* is a crucial first step towards identifying periods at high risk of transmission for the two economically important viruses they transmit.

## Background

Transmission of vector-borne diseases depends on the interaction between hosts (domestic and wild), vectors and pathogens within suitable environmental and climatic conditions [1]. The complex interplay of multiple factors, and their dependence towards extrinsic factors, mean that pathogen transmission is not constant and is often distributed over time and space [2]. Identifying when the transmission is suspected to occur facilitates disease surveillance, vector control implementation and communication to populations and stakeholders. To achieve this, the bionomics and population dynamics of each vector species is a starting point to investigate. Indeed, daily, seasonal or longer-term changes in meteorological and environmental features can impact their presence and abundance and therefore, the rate of transmission of pathogens they carry [1, 3, 4].

Biting midges of the genus *Culicoides* are vector species of economically important viruses affecting livestock [5, 6]. In Reunion Island, Indian Ocean, five *Culicoides* species are recorded with different altitudinal distributions: *C. bolitinos*, *C. enderleini, C. grahamii*, *C. imicola* and *C. kibatiensis* [7]. Since the 1980’s, clinical cases of two *Culicoides*-borne viral diseases, bluetongue (BT) and epizootic hemorrhagic diseases (EHD), are observed in sheep and cattle resulting in economic losses [8]. Epidemiological investigations highlighted that outbreaks of bluetongue virus (BTV) and epizootic hemorrhagic diseases virus (EHDV) occurred on annual cycles, with BTV being enzootic and EHDV epizootic [8–10]. Clinical cases are almost exclusively reported during or after the rainy season (November–March). This could be linked to *Culicoides* population dynamics and prompts us to identify the drivers of these dynamics to better understand patterns of disease transmission.

In poorly studied biological systems such as *Culicoides* species in Reunion Island, statistical models of population dynamics may facilitate understanding of relationships between climatic and environmental factors, and the known distribution of vectors [11–13]. In addition to highlighting the sensitivity of a species to particular factors [13], statistical models could (i) improve the characterization of the biology of a species; (ii) estimate vector occurrence at unsampled space and time point [14]; and (iii) help analyze *Culicoides* abundance with different ecological characteristics in the same model framework [15, 16].

Many studies have described the influence of meteorological and environmental parameters on *Culicoides* populations [11–13, 15–19], mainly in temperate areas. Among the five species recorded in Reunion Island, models were developed only for *C. imicola*. In Senegal [18], Morocco [11] and South Africa [12], this species was associated with rainfall, normalized difference vegetation index (NDVI), temperature, wind speed and percentage cover of water bodies. These studies also highlighted the role of humidity and host density as parameters that can regulate the presence or abundance of *Culicoides* in tropical environments.

Other studies conducted in the Mediterranean Basin included non-climatic parameters (land use, landscape and eco-climate) which improved the representation of the suitability of the habitats occupied by *C. imicola* and the accuracy of abundance prediction at a local scale [15, 19].

The aim of the present study was to describe and predict the temporal dynamics of all *Culicoides* species present in Reunion Island. Fortnightly catches were conducted on 11 farms during 26 months, and a library of climatic and non-climatic factors was built up. Mixed-effect negative binomial hurdle models were developed to predict the presence and abundance of *Culicoides* species and identify factors promoting or depressing populations.

## Methods

### Study sites

**Fig. 1:**
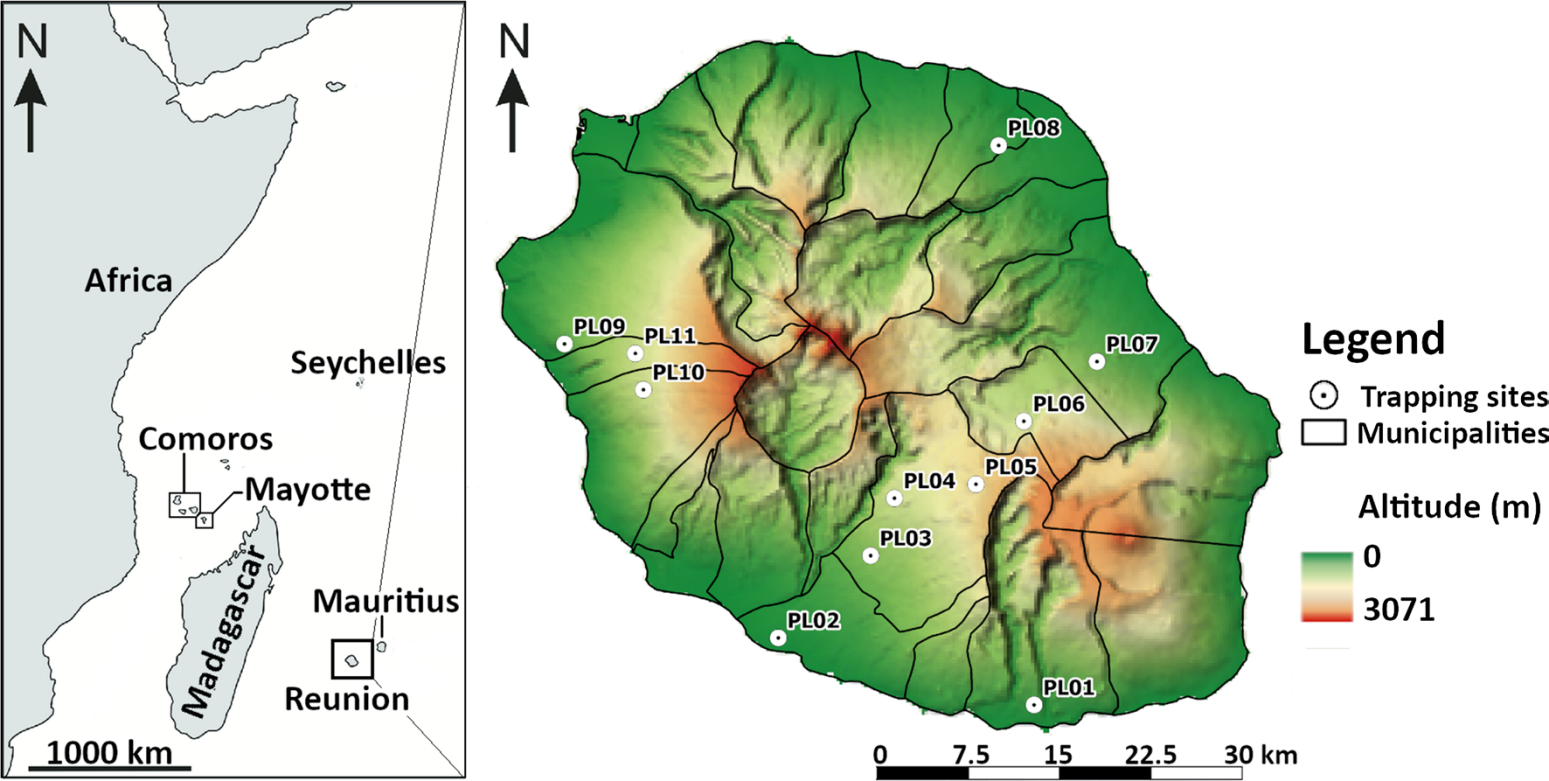
Map of the South-West Indian Ocean region localizing Reunion Island and the 11 study sites

Reunion Island, a French oversea department, is a mountainous (highest point 3069 m) small (2510 km^2^) volcanic island in the south-west of the Indian Ocean, 800 km east from Madagascar (Fig. 1). The island has a tropical wet climate with a warm rainy season (austral summer) observed from December to mid-April, and a cooler dry season (austral winter) between mid-April and November. The contrasted geography of the island strongly impacts the wind, rain and temperature patterns (Fig. 1). Winds mainly affect the eastern coast, bringing clouds which are blocked by the mountainous terrain. Thus, abundant rainfall (2000 to 8000 mm annually) is observed on the eastern coast, whereas the western coast only receives 600 to 2000 mm of rain per year [20]. Temperatures are closely linked with altitude. The mean annual temperatures range between 20.5–26 °C below 500 m; between 16 and 21 °C for altitudes from 500 to 1000 m; between 12–17 °C for altitudes from 1000 to 2000 m; and are ≤ 12 °C above 2000 m [20].

Livestock farming is well developed with several European improved breeds of ruminants like Prim’Holstein cattle for milk production, Limousine, Blonde d’Aquitaine cattle, Boer goats, Merinos and Romane sheep for meat production. According to the 2016–2017 census, the territory has approximately 16,000 cattle, 9000 goats and 2000 sheep (French Ministry of Agriculture and GDS Réunion). The mean holding of beef cattle, dairy cattle and sheep is 15, 41 and 67 animals per farm, respectively, in the structured sector. There are also 700 farmed Rusa deers (12 farms or game facilities), with up to 100 free-roaming deer in the highlands. Finally, there are 520 horses within 30 horse riding facilities.

Eleven sites were chosen among (i) farms with more than 10 animals; (ii) to cover the different geo-climatic regions and altitudes; (iii) and to coincide with cattle distribution because it is the dominant livestock species and because cattle are the most clinically impacted by EHDV and BTV infections [8, 10] (Fig. 1, Additional file 1: Figure S1). Cattle were present on all 11 sites. They were the only animal bred on six sites and they were bred alongside sheep or deer on the 5 other sites (Additional file 2: Table S1).

### *Culicoides* collection

Fortnightly catches were conducted from 12 January 2016 to 21 February 2018 using an Onderstepoort Veterinary Institute (OVI) black-light trap [21] in the 11 study sites. One site (PL04) could not be prospected for two months (from 6th September 2016 to 2nd November 2016) because of official sanitary regulations. Therefore, collection started on 15th November 2016 for the PL11 site with the opportunity to include a farm with deer at that time.

Traps were placed at 1.5 m to 1.8 m above the ground at the interface between inside and outside animal holdings or at the edge of the enclosure. Although light traps are only really efficient in the absence of sunlight, OVI traps were left in place and functional for 24 h (± 1h). It was a compromise between the logistical organization and the need to cover the activity of the *Culicoides* at each of the sites in a standardized way. For optimal preservation and identification, all insects were collected in water containing drop of liquid soap and transferred in 70% ethanol until their identification. All *Culicoides* were morphologically identified under a stereomicroscope to the species level based on a morphological identification key relevant for Reunion Island [7]. Large samples over 4 ml were sub-sampled to 3 ml of sedimented insect following a modified procedure described by Van Ark & Meiswinkel [22] to estimate the total number of *Culicoides*. Sex was also recorded.

### Meteorological and environmental data

Twelve categories of meteorological and environmental variables were used: temperature, humidity, rainfall, wind speed, global radiation, vegetation index, eco-climatic area, land use, farm density (i.e. the number of farms in 3 buffer areas defined around the trapping sites), animal density, length of watercourse and building opening size (Additional file 1: Figure S1, Additional file 2: Table S2 and Table S3).

Temperature and humidity were recorded every 4 h on site using thermo-hygro microchip recorder Hygrochip TH (Waranet Solutions Co., Auch, France) from 12th January 2016 to 22nd August 2017 and with Tinytag Plus 2-TGP-4500 Dual Channel temperature and RH data loggers (Gemini data loggers, Gemini Co., Chichester, UK) from 22nd August 2017 to 21st February 2018. Daily minimum, maximum and average temperature, minimum and maximum temperature between two trapping sessions and daily average humidity were extracted from the recorded data. Due to the failure of some recorders during the study, missing values were completed with Météo France and Cirad meteorological station data from the web platform https://smartis.re/HOME. Temperature data were automatically extrapolated at site coordinates using the web platform [23]. For humidity, data were extracted from the nearest meteorological station without modification. The replacement data, all before 22nd August 2017, represent between 11.6–75% of the data on sites PL02, PL04, PL05, PL07, PL08 and PL11, i.e. 26.5% of the humidity and temperature data for all sites combined. Despite taking into account the data from meteorological stations, 5% of the global humidity data was still missing.

Daily rainfall extrapolated at site locations, average wind speed at 2 m above the ground and global radiation from the nearest meteorological station during 24 h collection periods were also extracted from the web platform https://smartis.re/HOME. Data for wind and global radiation were sometimes not available (13.8% and 13.7%, respectively) during the study period. Some meteorological stations recorded wind speed only at 10 m above the ground. In that case, wind speed at 2 m above the ground was calculated by using the formula: $U2 = U10 \times { \ln }\left( {2/Z0} \right)/{ \ln }\left( {10/Z0} \right)$, where *U2* is the wind speed at 2 m above the ground, *U10* is the wind speed at 10 m above the ground and *Z0* is the roughness coefficient [24]. Categories of roughness of the land around trapping sites were estimate with the help of Atlas of Landscape and Roughness of Reunion Island (https://sites.google.com/site/venturiec1/calendar) and matched to coefficients according to ICAB® (https://www.icab.fr/guide/eurocode/en1991-1-4/).

The vegetation index was obtained from normalized difference vegetation index (NDVI) measurements at 250 m spatial resolution and 16 day frequency by the Moderate Resolution Imaging Spectroradiometer (MODIS) sensors (https://lpdaac.usgs.gov/) [25]. MODIS Terra 16-day composite images (product MOD13Q1) were acquired for the study period and the NDVI values were extracted for each site. Daily NDVI values were then estimated for each site using a temporal linear interpolation.

Eco-climatic area was extracted from the map of “Urban Planning and Native Plants Approach” (DAUPI) (Botanical Conservatory of the Mascarenes 2010; http://daupi.cbnm.org/palette/#/taxons). A land-use map [26] (http://aware.cirad.fr/layers/geonode%3Aclassif_gabir_2016_2017) was used to define 11 categories of land-use from which the percentage of land use categories were extracted in 0.5 km, 1 km and 2 km radius buffers around sites (Additional file 1: Figure S1). Size of buffers were based on estimated dispersal capacity of *Culicoides* [27, 28]. Hydrographical data from French National Geographic Institute (BD Topo® IGN; http://professionnels.ign.fr/bdtopo-hydrographie) were used to extract the length of watercourses flowing through the three buffer zones.

Farm and animal data were obtained by merging governmental and Groupement de Défense Sanitaire (GDS) de La Réunion (the main association of breeders of the island) census databases. Farm and animal densities were extracted for the three buffer sizes. Lastly, building opening size was recorded on the field. Production of maps and spatial analysis were performed using QGIS 2.18.20 software [29].

### Statistical analysis

All statistical analyses were performed using R version 3.4.1 [30]. Catch data collected before 22nd February 2016 were discarded because climatic data recorded on site of the previous months were not available for statistical analysis.

All missing values for humidity, wind and global radiation were estimated using the MissMDA package [31] which imputes the data by taking into account both the similarities between individuals and the links between variables using multiple factor analysis.

The dataset was split into two parts: (i) a training dataset, defined by a random selection of 2/3 of the dates from 22nd February 2016 to 21st February 2018 used to build statistical models; and (ii) a validation dataset, defined by the remaining third of the dates, used to estimate the predictive capacity of the models.

In order to consider nonlinear correlations between explanatory variables and *Culicoides* abundance, variables were log_10_-transformed, or reclassified into 3, 4 or 5 class quantiles when appropriate.

For each species, classes of categorical variables with low numbers of positive or negative catches per class were grouped. Values of NDVI, which range from 0 to 10,000, were rescaled from 0 to 100 in order to have a range similar to those of other variables.

To define the time lag with which variables were best correlated with observed *Culicoides* abundance, Cross Correlation Maps (CCM) analysis [32] was performed. CCM analysis was applied to minimum, maximum and average temperature, relative humidity, rainfall and vegetation index and their log_10_-transformation. For each variable and each species, a CCM analysis was performed using a timeframe of 50 days. If the lag giving the best correlation was a period between 50 and 30 days before the capture, a second lag within a timeframe of 30 days was also selected in order to take into account that the life-cycle of *Culicoides* species can be completed in less than a month under favorable conditions [6].

Mixed effect models were developed to consider the repeated nature of the data. Moreover, large numbers of zero counts are commonly observed with vector abundance data leading to zero inflation and over-dispersion in classical count regression models, such as Poisson regression [33]. Zero inflation and over-dispersion were verified using “zero.test” function in *vcdExtra* package [34] and “dispersiontest” function in the *AER* package [35], respectively. Considering repetition on site, and to deal with zero inflation and overdispersion, mixed effect negative binomial hurdle (NBH) models, with random effect on farms, were chosen to explain the abundance of each *Culicoides* species.

Hurdle models specify one process for zero counts and, once a threshold is crossed, another process for positive counts [33]. For the first step of the hurdle model, a mixed-effect logistic regression model (*lmer* package [36]) was developed to explain the presence (positive realization) *vs* absence (negative realization) (binary outcome) of each *Culicoides* species. If the species was present in a given site at a given time (i.e. if the realization is positive), the hurdle is crossed, and the conditional distribution of the positive is governed by mixed-effect zero-truncated negative binomial model developed using the *glmmTMB* package [37].

Both steps of the NBH model were developed by successively performing univariate and multivariate analyses, conducted separately for the binary and count (abundance) parts of the model. Variables with a *P-*value greater than 0.2 at the univariate level were not selected. To avoid duplication of information and promote the most informative meteorological and vegetation index variables, priority was given to numerical variables followed by categorical variables with the largest number of classes and then log_10_-transformed variables. This priority was also given to variables from the CCMs if the variable and its log_10_-transformation had the same lag. To avoid collinearity among remaining variables, generalized variance inflated factor (GVIF) using “vif” function in the *car* package [38] were computed on the fixed effect place in generalized linear models (GLMs) as suggested by Cheng et al. [39]. One by one, variables with the highest GVIF were deleted until the GVIF was less than 2, which indicates no collinearity.

At the multivariate analysis stage, an automatic stepwise selection, according to the Akaikeʼs information criterion (AIC), was used on the mixed logistic models. This automatic stepwise selection is based on a set of adding or dropping of each variable, from the current model, leading to the best AIC improvement (smaller AIC). Then, the null hypothesis of no random effect on these mixed logistic models was tested with *glmmML* package [40, 41] in 10,000 boot replicates. If the tests were significant for mixed logistic models for all species, random effects were not dropped out of the models. If the tests were not significant for all species, simple logistics models, maintaining the same structure of fixed effects, were applied to each species. Concerning mixed effect zero-truncated negative binomial models, as recommended by [39] for (generalized) linear mixed-effect models, a manual backward stepwise selection was used, also according to the AIC. There is, to our knowledge, no function that enables testing the significance of the random effect in zero-truncated negative binomial mixed models.

To combine each part of the NBH model, the threshold defining a zero or a positive realization was chosen to maximize the sensitivity and the specificity of the binary outcome on the ROC curve and using the *PresenceAbsence* package [42]. In other words, the chosen threshold minimized the mean of the error rate for positive observations and the mean of the error rate for negative observations. The fit of the final NBH models was estimated by AIC equal to [AIC_count_ × (1 – n > 0/n)] + AIC_binary_, where AIC_count_ and AIC_binary_, correspond, respectively, to the values of AIC for the count and binary model parts, and n and n > 0, correspond, respectively, to the total number of samples and the subset with positive counts only [43].

Then, predictive performances were assessed for the logistic model, count model and the final mixed effect NBH model. For the logistic model, the predictive accuracy was measured using the area under curve (AUC) of the ROC (Receiver Operating Characteristic) curve [44] with the *pROC* package [45]. For the count model and the NBH model, an Euclidean distance, the Normalized Root mean Square Error (NRMSE), was calculated using the *hydroGOF* package [46] by: $NRMSE = \sqrt {\left( {pred - obs} \right)^{2} /N} /\left( {obs_{max} - obs_{min} } \right)$; with *obs*, the observed abundance of the *Culicoides* sp., *obs*_*max*_ the maximum observed abundance, *obs*_*min*_ the minimum observed abundance, *pred*, the predictive values and *N* the number of observations. The NRMSE (expressed as a percentage if multiplied by 100) thus refers to the average error rate relative to the range of the observed values. The closer the NRMSE is to zero, the better the predictive performance. An internal validation was carried out on each part and on the final mixed effect NBH model using a leave-one-out cross-validation (LOOCV) using the *foreach* package [47]. LOOCV, also called n-fold cross-validation [48], minimizes the perturbation of the training data introduced during a cross-validation procedure [49]. An external validation was also conducted in which the predictive capacities of models were assessed using the validation dataset. The differences in AUC and NRMSE induced by internal and external validations reflect the reliability of the models: the smaller the differences, the more reliable the models.

## Results

**Fig. 2:**
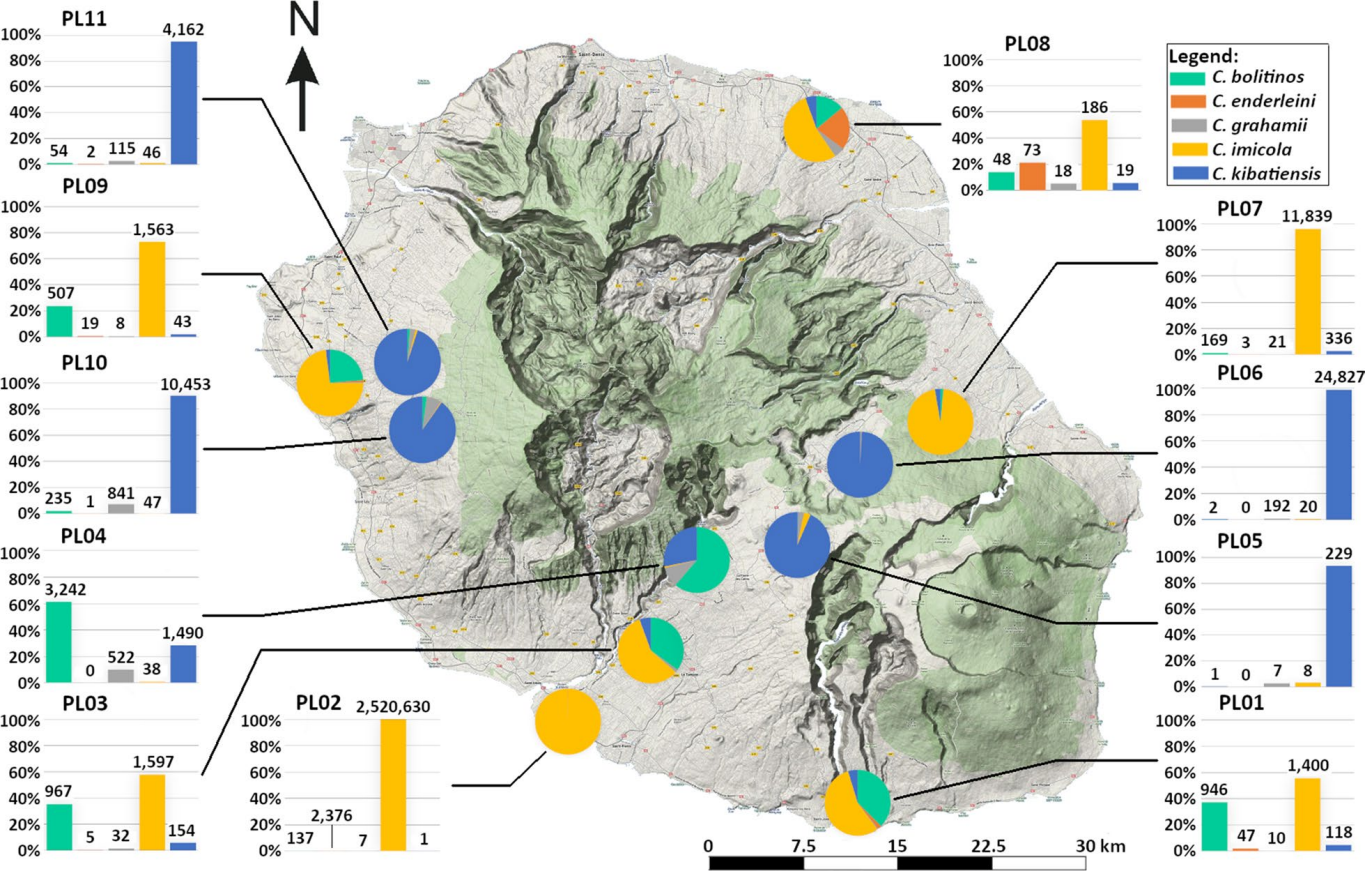
Diversity and total abundance at the study sites. Pie charts show the relative frequency of each species on site. Observed abundance resulting from 55 trapping sessions per site except for PL04 (49) and PL11 (33). Bar graphs: site number on title, relative frequency on y-axis, number of individuals on bar labels. Topographical map from French National Geographic Institute (BD Topo® IGN; http://professionnels.ign.fr/scan-ign)

A total of nearly 2.6 million biting midges were collected during 577 night-catches at 11 locations and over 26 months, from January 2016 to February 2018 (Table 1, Fig. 2). This large-scale and extensive survey confirmed the presence of the five *Culicoides* species recorded in 2005 [7]. Overall, 48 night-catches (8.3%) did not collect any *Culicoides*. Total observed abundance over the collection period varied greatly among sites (Table 1, Fig. 2): 245 and 344 individuals for PL05 and PL08, respectively (for the entire collection period), compared to 2.5 million for PL02. In other sites, the number of *Culicoides* caught ranged from 2100 to 25,040 individuals. The total number of individuals and percentage of positive catches (in parentheses) per species were: 2,537,374 (57.9%) for *C. imicola*; 41,832 (56.8%) for *C. kibatiensis*; 6310 (42.8%) for *C. bolitinos*; 1773 (29.5%) for *C. grahamii*; and 2526 (12.5%) for *C. enderleini*. Thus, mean number of individuals per positive catch was 7597 for *C. imicola*, and ranged from 10.42 for *C. grahamii* to 127.1 for *C. kibatiensis*. *Culicoides imicola* was mostly collected from a single site (PL02: 99.34% of total catches, 45,829 individuals/positive catch). Not considering this site, only 16,744 (52.3%) *C. imicola* were collected from the 10 other sites in 522 catches (60.01 individuals/positive catch on average).

**Table 1:** Number of *Culicoides* caught and percentage of positive catches on the 11 sites in Reunion Island

Site (*N*)	*C. bolitinos*			*C. enderleini*			*C. grahamii*			*C. imicola*			*C. kibatiensis*			Total *Culicoides spp.*		
	n	Pos (%)	Mean ± SD	n	Pos (%)	Mean ± SD	n	Pos (%)	Mean ± SD	n	Pos (%)	Mean ± SD	n	Pos (%)	Mean ± SD	n	Pos (%)	Mean ± SD
PL01 (55)	946	67.3	17.2 ± 31.82	47	20.0	0.85 ± 2.96	10	12.7	0.18 ± 0.55	1400	81.8	25.45 ± 81.71	118	34.5	2.15 ± 4.79	2515	89.1	9.17 ± 40.35
PL02 (55)	137	10.9	2.49 ± 8.79	2376	50.9	43.2 ± 99.8	7	1.8	0.13 ± 0.94	2,520,630	100.0	45,830 ± 69,606	1	1.8	0.02 ± 0.13	2,522,564	100.0	9175.1 ± 35,944
PL03 (55)	967	80.0	17.58 ± 28.8	5	7.3	0.09 ± 0.35	32	32.7	0.58 ± 1.08	1597	96.4	29.04 ± 52.8	154	54.5	2.80 ± 6.04	2755	96.4	10.02 ± 29.2
PL04 (49)	3242	87.8	66.2 ± 127.5	0	0.0	0 ± 0	522	67.3	10.65 ± 31.4	38	30.6	0.78 ± 1.49	1490	79.6	30.41 ± 40.5	5291	95.9	21.61 ± 65.9
PL05 (55)	1	1.8	0.02 ± 0.13	0	0.0	0 ± 0	7	9.1	0.13 ± 0.43	8	9.1	0.15 ± 0.52	229	61.8	4.16 ± 7.62	245	69.1	0.89 ± 3.77
PL06 (55)	2	1.8	0.04 ± 0.27	0	0.0	0 ± 0	192	36.4	3.49 ± 6.88	20	9.1	0.36 ± 1.5	24,827	100.0	451.4 ± 490.2	25,040	100.0	91.06 ± 282.7
PL07 (55)	169	32.7	3.07 ± 12.05	3	5.5	0.05 ± 0.23	21	16.4	0.38 ± 1.25	11,839	100.0	215.25 ± 197.1	336	67.3	6.11 ± 7.96	12,363	100.0	44.97 ± 122.4
PL08 (55)	48	21.8	0.87 ± 3.0	73	32.7	1.33 ± 2.37	18	12.7	0.33 ± 1.2	186	47.3	3.38 ± 12.47	19	12.7	0.35 ± 1.21	344	67.3	1.25 ± 5.95
PL09 (55)	507	72.7	9.22 ± 20.13	19	20.0	0.35 ± 0.89	8	10.9	0.15 ± 0.45	1563	90.9	28.42 ± 41.6	43	41.8	0.78 ± 1.23	2100	94.5	7.78 ± 23.25
PL10 (55)	235	56.4	4.27 ± 8.53	1	1.8	0.02 ± 0.13	841	80.0	15.29 ± 29.6	47	25.5	0.85 ± 2.56	10453	96.4	190.05 ± 315.7	10,830	100.0	42.1 ± 159.2
PL11 (33)	54	48.5	1.64 ± 2.61	2	3.0	0.06 ± 0.35	115	63.6	3.48 ± 5.15	46	33.3	1.39 ± 2.77	4162	100.0	126.12 ± 136	4339	100.0	26.54 ± 78.2
All sites (577)	6410	43.2	10.94 ± 43.8	2526	13.3	4.38 ± 33.1	1773	29.6	3.07 ± 14.0	2,537,374	57.9	4397.5 ± 25,207	41832	57.4	72.5 ± 227.4	2,589,815	91.7	897.7 ± 11,401

*Abbreviations*: N, number of night-catches; n, number of *Culicoides*; Pos (%), percentage of positive catches; Mean, mean number of *Culicoides* at all trapping sessions; SD, standard deviation

The five species were caught at least once in 8 of the 11 sites. A strong spatial heterogeneity was observed for abundance (Fig. 2). *Culicoides imicola* was the most abundant species at the 6 sites with the lowest altitude (i.e. less than 660 m) where it represented more than 95% of the catches in PL02 and PL07 and more than 50% in the 4 others (PL01, PL03, PL08 and PL09). *Culicoides kibatiensis* was the most abundant at 4 of the 5 sites (PL05, PL06, PL10 and PL11) with the greatest altitude (i.e. greater than 1195 m) where it represented more than 95% of the catches. *Culicoides bolitinos* was abundant in sites with altitudes ranging from 200 to 1200 m and represented more than 50% of the catches in PL04.

When merging all sites together, dynamics of the five species showed strong differences (Additional file 3: Figure S2). Three species, *C. bolitinos*, *C. enderleini* and *C. grahamii*, showed a decrease in abundance from July to November 2016, corresponding to the cold and dry season. However, this decrease in abundance was not observed in 2017 for *C. bolitinos* and *C. grahamii*. The two other species, *C. imicola* and *C. kibatiensis*, did not display such seasonal decreases in abundance during the observation period.

**Fig. 3:**
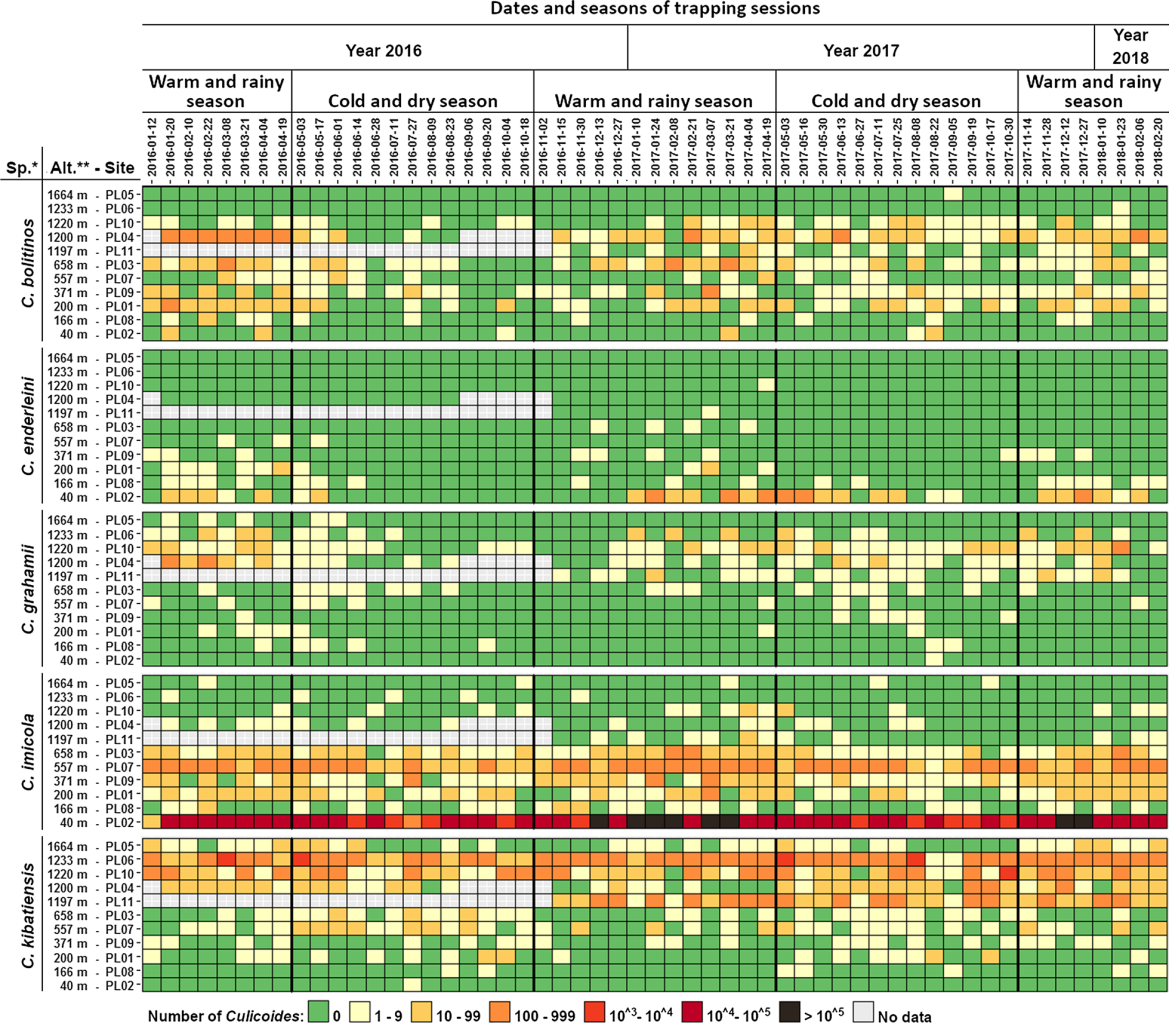
Mondrian matrix of fortnightly *Culicoides* abundance (color coded) of the five species over the two years of trapping collection (January 2016–February 2018) and at the 11 trapping sites. *Key*: *sp., species; **Alt., altitude in meters. Bold vertical lines separate the seasons. Note that the sites are classified by increasing altitude

Differences in dynamics were also observed for each species between sites (Fig. 3, Additional file 3: Figure S2). Without considering sites with no positive catches for a given species, three types of dynamics were observed: (i) continuous if no more than two sequential zero catches were observed; (ii) sporadic if each positive catch was separated by more than 2 zero catches; and (iii) seasonal for intermediate situations. Thus, the continuous dynamics of *C. imicola* and *C. kibatiensis* were observed only in 4 (PL02, PL03, PL07 and PL09) and 3 (PL06, PL10 and PL11) sites, respectively. Seasonal dynamics were observed in the other sites except in PL05 and PL06 for *C. imicola* and in PL02 for *C. kibatiensis*. *Culicoides bolitinos* and *C. grahamii* showed seasonal dynamics on each site except for PL05 and PL06 for the former and PL02 for the latter. Last, seasonal dynamics were observed for *C. enderleini* in 6 sites (PL01, PL02, PL03, PL07, PL08 and PL09).

Detailed information on the covariates associated with the species and from final mixed-effect NBH models (coefficients and their standard errors, odds ratios (OR) or incidence rate ratios (IRR), their 95% confidence intervals and the value of t-statistic) are presented in Additional file 4: Tables S4–S8. As site PL02 represented 99.34% of all *C. imicola* caught, we considered two datasets to model *C. imicola* abundance: one with all sites, and one without the outlier site (PL02). Statistical results for the latter are presented in Additional file 4: Table S9, but are not discussed here. In addition, no significant random effects were detected for the logistical part of the NBH models for all species, including *C. imicola* without PL02. Each NBH model therefore consists of a simple logistic model to estimate presence/absence and a mixed effect zero-truncated negative binomial model to estimate abundances. The thresholds defining a zero or a positive realization for each species were as follows: 0.555 for *C. bolitinos*; 0.2 for *C. enderleini*; 0.27 for *C. grahamii*; 0.61 for *C. imicola*; and 0.58 for *C. kibatiensis*. Specificity varied between 0.793–0.929 and sensitivity between 0.736–0.870.

**Fig. 4:**
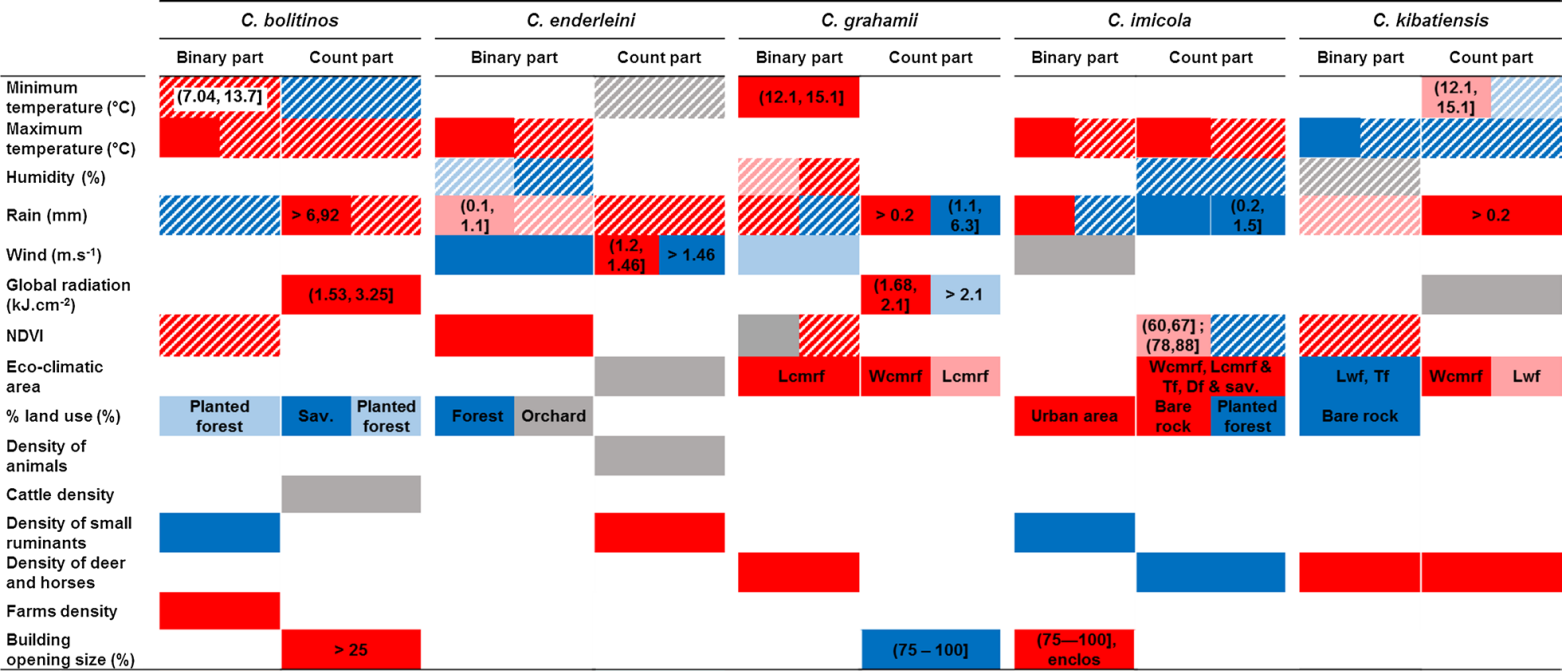
Matrix of correlation between meteorological-environmental variables and presence-abundance of each *Culicoides* species in the final mixed-effect negative binomial hurdle model. Red, significant (*P* < 0.05) favorable effect on presence or abundance of the *Culicoides* species; blue, significant (*P* < 0.05) unfavourable effect; stripped, lagged effect of the variable on catch; light colours, less significant effect (*P* < 0.1); grey, variables retained by the model but without significant effect. For categorical variables, modalities associated with the correlation are represented. For reference modalities in categorical variables and detailed information, see Additional file 4: Tables S4–S8. *Abbreviations*: Lcmrf, leeward coast mountain rain forest; Wcmrf, windward coast mountain rain forest; Lwf, lowland wet forest; Tf, tamarind forest; Df, dry forest; Sav., savannah

Regarding correlations with meteorological and environmental variables (Fig. 4), four main categories of variables impacted presence and/or abundance of all species: rain, temperature, NDVI and host densities. To a lesser extent, a 5th category related to a forested environment (as described by land use and eco-climatic areas) also impacted the presence and abundance of the five species. Although some parameters impacted all five species, their importance, based on the absolute value of t-statistic (Additional file 4: Table S4–S8), varied.

Rain was the only explanatory variable that affected both presence and abundance of all five species. Rain had a positive effect on all species but could be offset by a negative effect depending on the quantity and the period considered in relation to the catch. For example, rain at trap retrieval had a positive effect on the presence of *C. imicola*, but a negative effect if it rained 4 days before trapping. Rain was the most important driver of the abundance of *C. grahamii* and *C. kibatiensis*. Rain at trap retrieval between 1.1–6.3 mm negatively affected the former and, in contrast, rain at trap setting between 1.5–6.9 mm positively affected the latter.

Temperature had an effect on presence of all species and on abundance of some species (*C. bolitinos*, *C. imicola* and *C. kibatiensis*). It was the most important driver of presence of *C. kibatiensis* and abundance of *C. bolitinos.* This driver impacted the former negatively and with a lag of time between two trapping sessions. The latter was positively impacted with a lag of 35 days. In addition, temperature was the second most important and positive driver of the presence of *C. bolitinos*, *C. enderleini* and *C. imicola*.

NDVI was positively associated with all species, except *C. imicola*. It was one of the most important parameters affecting the presence of *C. grahamii* with a lag of 31 days.

Another category of covariates associated with all species was host density. The most important factors governing the presence of *C. bolitinos* and the abundance of *C. enderleini* were the density of small ruminants in 0.5 and 1 km radius, respectively. For *C. grahamii*, density of deer and horses in 2 km radius was the second most important parameter affecting positively its presence.

Last, forested environment was also associated with all species. The presence and/or abundance of *C. grahamii*, *C. imicola* and *C. kibatiensis* were strongly and positively associated with eco-climatic areas such as leeward and/or windward coast mountain rain forest. *Culicoides imicola* was also positively associated with dry forest and savannah.

The other parameters were not associated with all species. The presence of *C. enderleini* was negatively governed by wind speed at trap setting (most important factor). Similarly, building opening size was the most important parameter explaining the presence of *C. imicola*.

**Fig. 5:**
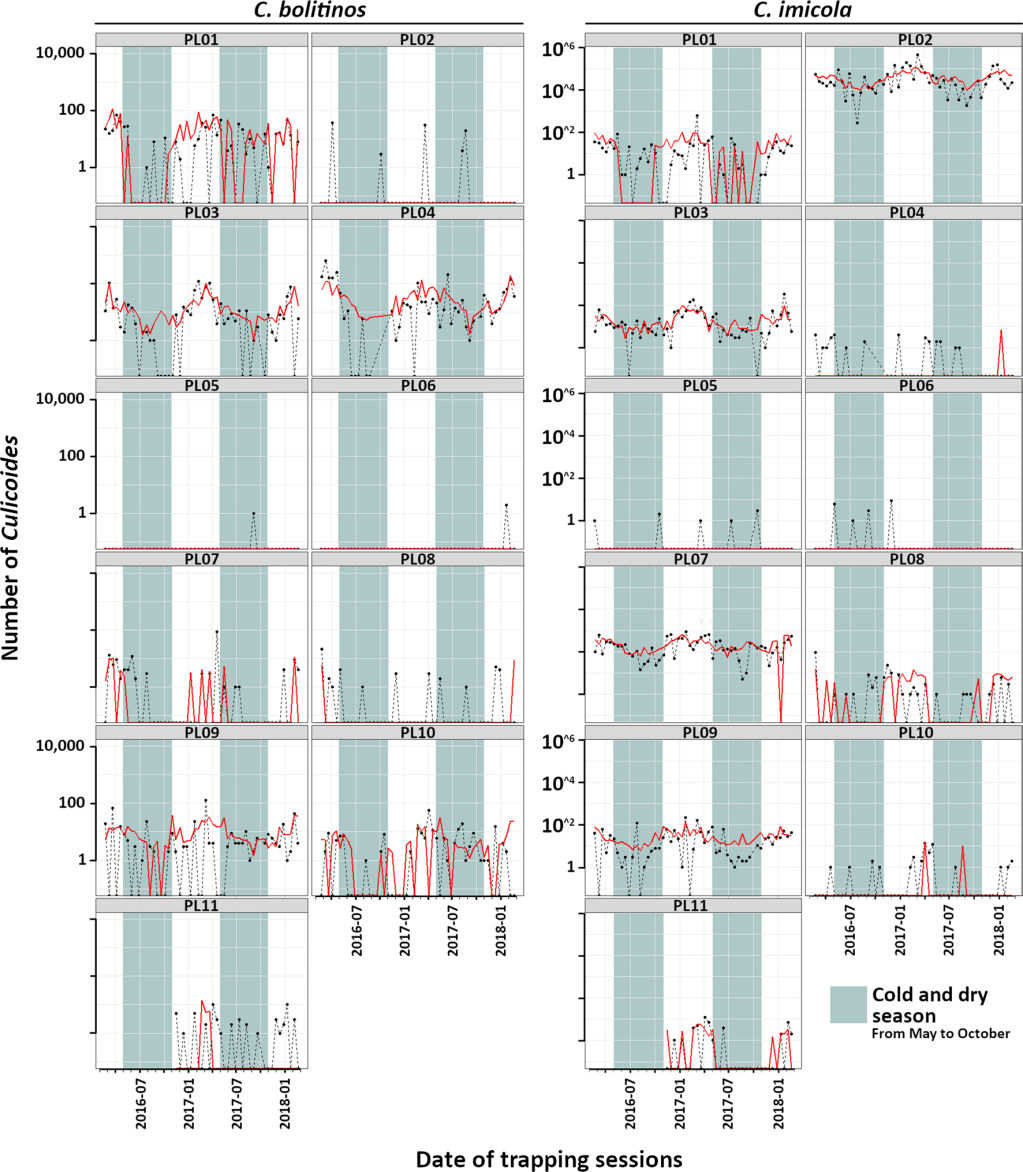
Prediction graphs of *C. bolitinos* and *C. imicola* abundance according to the final mixed effect negative binomial hurdle modelling by site. Black and dashed line, observed abundance; red line, predicted abundance. Note the log_10_ scale for the number of *Culicoides*

The comparison of the predictions of the models and the observed *Culicoides* abundances (Fig. 5 for *C. bolitinos* and *C. imicola*, the two proven vectors of BTV and EHDV [50, 51]; Additional file 5: Figure S3 for the three other species), showed that the mixed effect NBH models correctly predicted the seasonal variation in abundance, as well as difference in abundance level between sites.

Overall, the predictive accuracy of presence and absence of each species were good or excellent: ROC AUCs varied from 0.884 to 0.949 (Additional file 4: Table S10). Results of internal validation by LOOCV showed that predictive performances remained globally similar, with slight decreases of AUCs of -0.015 to -0.034. External validation results showed that the predictive performance was acceptable for *C. grahamii* (AUC = 0.743) and good for other species (AUCs ∈ [0.813, 0.847]). For the count part of the model, the NRMSEs varied from 9% to 17%. The best predictive performance was observed for *C. imicola* and *C. kibatiensis* with NRMSEs of 9% and 11.3% respectively.

Finally, for the complete mixed effect NBH models, NRMSEs varied from 4.4% to 8.5%. The best predictive performance was observed for *C. enderleini* (4.4%). Remarkably, external validation yielded better results for *C. bolitinos*. Indeed, the variation was -0.9%, reflecting an improvement in the error rate. For *C. enderleini*, the value of NRMSE varied from 4.4% to 17.6% during the external validation, i.e. a 4-fold increase in the error rate. For *C. grahamii*, *C. imicola* and *C. kibatiensis*, NRMSEs were 4.9%, 4.8% and 5.9%, respectively. During external validation, their NRMSEs increased by 6.7%, 1.3% and 4.8%, respectively.

## Discussion

This study is the largest sampling effort ever carried out on Reunion Island for *Culicoides* biting midges associated with livestock using reference traps, while comprehensively examining the relationship between seasonal population dynamics and meteorological and environmental conditions.

The survey confirmed the low specific diversity (five species) [7] compared to Mayotte, a 6-fold smaller volcanic island (374 km^2^) belonging to the Comoros Archipelago (Fig. 1), where a recent inventory listed at least 17 species including *C. bolitinos*, *C. enderleini*, *C. imicola* and *C. kibatiensis* [52]. This relatively low diversity in Reunion Island could be linked to its greater geographical remoteness (684 km from Madagascar and 1680 km from the African coastline as opposed to 295 and 485 km for Mayotte, respectively). Diversity in the other islands of the Indian Ocean have not yet been thoroughly assessed. Only two species (*C. imicola* and *C. enderleini*) sampled in three sites were described in Mauritius [53]. In Seychelles, two limited inventories listed three species [54, 55] not found in Reunion Island. In the other islands of the Comoros Archipelago, no inventory has been carried out. Finally, in Madagascar, few species, including *C. imicola* and *C. enderleini*, have been described [56, 57] but the inventory remains largely incomplete.

Except for one site (PL02), observed *Culicoides* abundance on the island was low compared to other areas in the Afrotropical region where the same trap was used and where average catches were in the hundreds to tens of thousands of individuals (in South Africa [12, 58–60], Zimbabwe [61] and Senegal [62]). These low abundances could be explained by a lack of favorable microhabitats [63] due to the presence of volcanic soil, and/or sub-optimal climate for the development and survival of *Culicoides* [64].

Exceptionally, high numbers of *C. imicola* were caught in site PL02 all year round. Local variations in abundance are well described for *Culicoides* in different areas [11–13, 21, 61, 62, 65–67]. Variations in climate, environment, host density, and farming practices are usually advocated to explain the observed differences [17, 68, 69]. Indeed, unlike the other sites, PL02 has environmental and climatic characteristics closer to those recorded in the Afrotropical region where *C. imicola* is frequently found and in high abundance: dry vegetation, dry winter, favorable year-round temperature, hot summer, and presence of mammalian hosts [58, 61, 62].

Three of the five species recorded (*C. bolitinos*, *C. enderleini* and *C. grahamii*) showed marked seasonality with a total abundance decrease during the cold and dry season whereas the two most abundant species (*C. imicola* and *C. kibatiensis*) showed continuous dynamics during the 26 months follow-up. However, strong variations of dynamics were observed at a finer scale, i.e. between sites, for each species.

Temperature appears to be the main seasonal factor for *Culicoides* in Reunion Island, with a clear positive impact on the presence and abundance of *C. imicola* and *C. enderleini*, and a negative impact for *C. kibatiensis*. The temperature seemed also favorable to *C. bolitinos* but with a limiting effect above a threshold (i.e. when temperatures are too high), thus explaining why this species was less abundant at low altitude. Concerning *C. grahamii*, its relationship to temperature is less obvious here because its distribution is associated with cold temperatures whereas its seasonality seems to indicate a positive impact of higher temperatures on population size. Interestingly, for *C. bolitinos* and *C. grahamii*, a clear decrease in total abundance was not observed during the 2017 cold and dry season compared to 2016. According to the climate monitoring of Météo-France on Reunion Island (http://www.meteofrance.re), the cold and dry season of 2017 was warmer and wetter than that of 2016. These different temperature and rainfall patterns in 2017 could have extended the favorable weather period for these species, thus underlining that the dynamic of a species can vary from one year to the next.

Eleven of the twelve categories of meteorological and environmental variables tested (all variables except length of watercourses) were linked to the presence and/or abundance of at least one species of *Culicoides*. Moreover, a same driver could affect the same species in several ways: (i) in an ambivalent way, such as rain, which had both a positive and a negative effect on *C. grahamii* for example; and (ii) during capture and/or with one or more lagged effects, undeniably suggesting impacts that could affect adults but also larval stages. The correlations with earlier dates can thus provide information on when these species are most sensitive to climatic hazards during their life-cycle and potentially when certain immature phases occur.

Many studies have associated *C. imicola* with various climatic and environmental factors [11, 12, 15, 16, 18, 70–72]. According to our results, the behavior and preference for a habitat type were the first characteristics to consider for this species. Its strong association with widely open buildings or enclosures underlines the importance of its exophilic behavior as observed by Banard & Meiswinkel et al. [73, 74]. With regard to the relationship of this species with the different climatic zones of the island and secondarily with land use (forest), the study confirms its preference for a dry vegetation and/or areas with low vegetation that are exposed to the sun [15, 19, 58, 62, 67]. The association between NDVI and *C. imicola* further supports this result. Moreover, such a long lag (50 days) in the impact of NDVI could reflect a long-term impact of vegetation on *C. imicola* population. Unfavorable conditions can reduce the quality of larval habitats and thus reduce adult recruitment. The effect of temperature and rain on flight activity, mortality or life-cycle speed of *C. imicola*, and *Culicoides* in general, has been widely described [5, 6, 18, 71, 75–81]. The presence of hosts is also a key factor affecting *Culicoides* populations [13, 18, 82, 83]. However, our results are very surprising about the link between *C. imicola* and the density of small ruminants or deer and horses. With regard to models associating *C. imicola* with a host type [70, 71] or studies on its trophic preference [84–87], none allow us to establish hypotheses. Thus, the question “how can a higher abundance of small ruminants, horses or deer in the surroundings of a cattle farm reduce the abundance of *C. imicola* in that farm?” remains unanswered.

Compared to *C. imicola*, the relationship between *C. bolitinos* and the climatic and environmental drivers showed tangible differences. Our results suggest that *C. bolitinos* was greatly affected by the quantity, quality and viability of its larval habitat, as reported by Meiswinkel et al. [88] in South Africa. The strong association of this species with farm and small ruminant density can be linked, respectively, to the quantity and quality of available larval habitats. Also, the strong and multiple delayed effects of temperature, followed by those of rain and NDVI, seem to define the viability of these larval habitats. Induced modulations on the intrinsic characteristics of the breeding site (temperature, moisture) or leaching could impact immature stages [5, 6, 89, 90]. It is interesting to note that the positive effect of NDVI on *C. bolitinos* associates this species with wetter, and therefore greener, environments [91] than those of *C. imicola*. With regard to the association with the building opening size, *C. bolitinos* exhibited a greater endophilic behavior than *C. imicola.* This result is also comforted by Meiswinkel [74] who showed that numbers of *C. bolitinos* dropped if entry size in buildings was too small. *Culicoides bolitinos* also showed a rather complex relationship with rain. Perhaps the fresh and humid conditions after a rainfall favor its activity. Last, the effect of global radiation on *C. bolitinos*, which can affect several parameters such as temperature, evapotranspiration or biomass production [92, 93], was probably a marker of seasonality.

Like *C. imicola*, the association of *C. enderleini* with temperature and forest suggests a preference for hot and sparsely vegetated habitats exposed to full sunlight, which is consistent with observations made in Senegal [62, 94] and Gambia [95]. However, the abundance of *C. enderleini* was most impacted by the density of small ruminants. In South Africa [96], this species was found near cattle with confirmed blood meal sources on horses and sheep, which underlines the importance of hosts for this species. Interestingly, *C. enderleini* was the only species significantly affected by wind. Wind speed can reduce dramatically the *Culicoides* activity [5, 97] but Jess et al. [98] suggested that the relation to wind speed may not be linear. Indeed, the flight of some insects can be stimulated by low winds [99]. This seems to be the case for *C. enderleini,* who was favored by wind speeds of 1.2–1.5 m/s and inhibited by higher wind speeds. Finally, the results showed lag effects of rain and humidity. These lags seem to indicate an effect on larval development, but the underlying ecological reasons explaining theses associations remain difficult to clarify. *Culicoides enderleini* was also associated with NDVI, which is clearly a marker of seasonality [15], reflecting also seasonal rainfall [100].

*Culicoides kibatiensis*, the second most abundant species on the island, often exhibited associations with climatic or environmental parameters contrary to those of *C. imicola*. First, its association with temperature confirms that *C. kibatiensis* is a cold-adapted species and suggests that the survival of immature stages, and potentially the fertility of the previous generation [80] prevail over the speed of the life-cycle. Concerning rainfall, *C. kibatiensis* was more abundant at high altitude, where the cloud cover is formed. The induced mist can lead to a form of precipitation that will be recorded by meteorological stations and this could explain the link between *C. kibatiensis* and light rainfall. Secondly, the strong relationship of this species with mountain rain forest eco-climates reinforces its association with cold environments and underlines its preference for rather humid and high-altitude climates in Reunion Island. This observation differentiates it even more from *C. imicola*, as well as from *C. enderleini*. Thirdly, the presence and the abundance of *C. kibatiensis* were positively associated with deer and horses. This seems plausible as a large proportion of *Culicoides* species are opportunistic in host selection and several livestock-associated mammophilic species are known to feed on wild deer [6]. Finally, and similarly to *C. bolitinos*, the effect of vegetation suggests a preference for wetter and greener environments for larval habitats than *C. imicola*.

The least abundant species, *C. grahamii*, was associated with mountain rain forest eco-climatic areas, suggesting, as for *C. kibatiensis*, a preference for rather humid and high-altitude climates. This preference was secondarily supported by the effect of humidity, NDVI and minimum temperature of 12–15 °C on this species. The first two conditions (humidity and NDVI) support the hypothesis that wetter conditions favor breeding sites. The last could correspond to the optimal temperature range for the activity of this species, as shown by Venter et al. [75] for *C. imicola*. *Culicoides grahamii* also showed a complex relationship with rain, the most influential parameter affecting its abundance. It can be hypothesized that the peak activity at dawn [101] may be particularly constrained by rain whereas rainy weather during the day could allow this species to have a daytime activity as observed by Nicholas et al. [102]. Concerning the lagged effect of rain on the presence of this species, one can hypothesize an impact on the availability of the breeding sites, i.e. small bodies of rainwater with a muddy bottom and which are rich in organic matter [103]. Finally, and conversely to *C. imicola* and *C. bolitinos*, *C. grahamii* seems to have an endophilic behavior. This species is very small and could search for protection again climatic hazards given its size [7].

Many studies have shown that the inclusion of non-dynamic variables with dynamic variables increases the fit of models [15, 17, 83, 89, 104–106]. Significant associations were highlighted between *Culicoides* species presence or abundance and landscape [19], soil type [105] and farm-associated variables such as breeding practices effluent management, hygienic measures, animal movements, use of insecticides and/or grassland mowing [107–110]. Our model fit suggests that overall no major driver of dynamics was omitted. Fortunately, when measurements are repeated in a sample, mixed models allow for farm-induced long-term differences to be taken into account. However, if these random effects at farm-level are not fully elucidated, it is not possible to extrapolate the models to other sites. Concerning our hurdle models, only the part concerning the estimation of abundance (count part) was affected by this restriction.

The statistical models developed in this study link presence and abundance of *Culicoides* to climatic and environmental factors with different time lags, suggesting an impact on the different stages of their life-cycle. Mechanistic models allow the different processes underlying population dynamics to be taken into account by compartmentalizing the different life stages. However, such models require knowledge on the mechanisms related to the system under study and on their drivers. As these were the first models developed for four of the five species (all except *C. imicola*) and the first models concerning the dynamics of *Culicoides* in Reunion Island, it was important to first develop statistical models in order to refine our knowledge on the drivers of dynamics of each species. CCM [111] proved very useful to identify the best lags for each dynamic variable, in turn helping identify on which stages the variables were impacting. By contributing to significantly improve our knowledge on the dynamics of *Culicoides* in Reunion Island, this work enables the further development of mechanistic models.

## Conclusions

To our knowledge, this study is the first to describe and model longitudinal data on *Culicoides* from Reunion Island. It confirmed the presence of five species, in relatively low abundance except for one species in one site (*C. imicola* in PL02), and presented their seasonal abundance patterns, giving more insight into the dynamics of *Culicoides* populations in the Afrotropical region. Through the identification of environmental and meteorological drivers of presence and abundance of the five *Culicoides* species, it improves our understanding of their ecology. *Culicoides imicola* dynamics was closely related to temperature and rainfall and it favored by dry open vegetation. *Culicoides grahamii*, *C. bolitinos* and *C. enderleini* showed marked seasonal patterns with a drop in abundance during the cold dry season. *Culicoides grahamii*, the least abundant species, showed relations with temperature similar to *C. imicola.* Results suggest it was more endophilic than *C. bolitinos* and *C. imicola*. As for *C. imicola*, *C. bolitinos* was positively associated to temperature and had more complex associations with rainfall but was associated to greener and wetter environments than *C. imicola*. *Culicoides kibatiensis* was clearly an outlier, abundant at high altitude, it was a cold-adapted species. *Culicoides enderleini* was positively associated to temperature and rainfall but negatively to humidity and had the particularity to be the only species significantly affected by wind. As at least two of the five species (*C. imicola* and *C. bolitinos*) are known vectors of orbiviruses to ruminants, this study is a first step to enhance ecological understanding of the dynamic nature of pathogen-vector-host interactions in *Culicoides*-borne virus systems. Further work should include assessing the vector roles of the three other species (*C. enderleini*, *C. kibatiensis* and *C. grahamii*) and developing spatially explicit models of dynamics and of pathogen transmission for the entire island. These spatially explicit dynamic models will help farmers and animal health stakeholders identify areas and periods of vector activity and at high risk of viral transmission.

## Supplementary information

**Additional file 1: Figure S1.** Temporal variation of dynamics variables over the two years of trapping collection (January 2016–February 2018) at the 11 trapping sites and maps of non-dynamics variables.

**Additional file 2: Table S1.** Characteristics of trapping sites. **Table S2.** Temporal variables considered on mixed-effect negative binomial hurdle models. **Table S3.** Non-dynamic variables considered on mixed-effect negative binomial hurdle models.

**Additional file 3: Figure S2.** Temporal variation of the fortnightly *Culicoides* abundance of the five species over the two years of trapping collection (January 2016–February 2018) and at the 11 trapping sites.

**Additional file 4: Table S4.** Final mixed-effect negative binomial hurdle model of risk factors associated with the count of *C. bolitinos*. **Table S5.** Final mixed-effect negative binomial hurdle model of risk factors associated with the count of *C. enderleini*. **Table S6.** Final mixed-effect negative binomial hurdle model of risk factors associated with the count of *C. grahamii*. **Table S7.** Final mixed-effect negative binomial hurdle model of risk factors associated with the count of *C. imicola*. **Table S8.** Final mixed-effect negative binomial hurdle model of risk factors associated with the count of *C. kibatiensis*. **Table S9.** Final mixed-effect negative binomial hurdle model of risk factors associated with the count of *C. imicola* without PL02 site. **Table S10.** Measurement of model fit (AIC), predictive accuracy or (AUC) and predictive performance (NRMSE) of the mixed effect negative binomial hurdle model for each species and each validation steps.

**Additional file 5: Figure S3.** Prediction graphs of *C. enderleini*, *C. grahamii* and *C. kibatiensis* abundance according to the final mixed effect negative binomial hurdle modelling by site.

**Additional file 6.** R codes hurdle models. R codes for the realization of hurdle models on the 5 *Culicoides* species present in Reunion Island.

## Data Availability

Data supporting the conclusions of this article are included within the article and its additional files. All samples and R codes are available upon request.

## Abbreviations

AIC: Akaike’s information criterion
AUC: area under curve
BT(V): bluetongue (virus)
CCM: cross-correlation map
DAUPI: urban planning and native plants approach
EHD(V): epizootic haemorrhagic disease (virus)
GDS: groupement de défense sanitaire
GLM: generalized linear model
GVIF: generalized variance inflated factor
LOOCV: leave-one-out cross-validation
MODIS: moderate resolution imaging spectroradiometer
NBH: negative binomial hurdle model
NDVI: normalized difference vegetation index
NRMSE: normalized root mean square error
OVI: Onderstepoort Veterinary Institute
ROC: receiver operating characteristic
