# Supplementary figures and images for: Modelling temporal dynamics of Culicoides Latreille (Diptera: Ceratopogonidae) populations on Reunion Island (Indian Ocean), vectors of viruses of veterinary importance
Source: Parasit Vectors. 2019 Nov 27;12:562. doi: 10.1186/s13071-019-3812-1 (PMC6880491; doi:10.1186/s13071-019-3812-1)

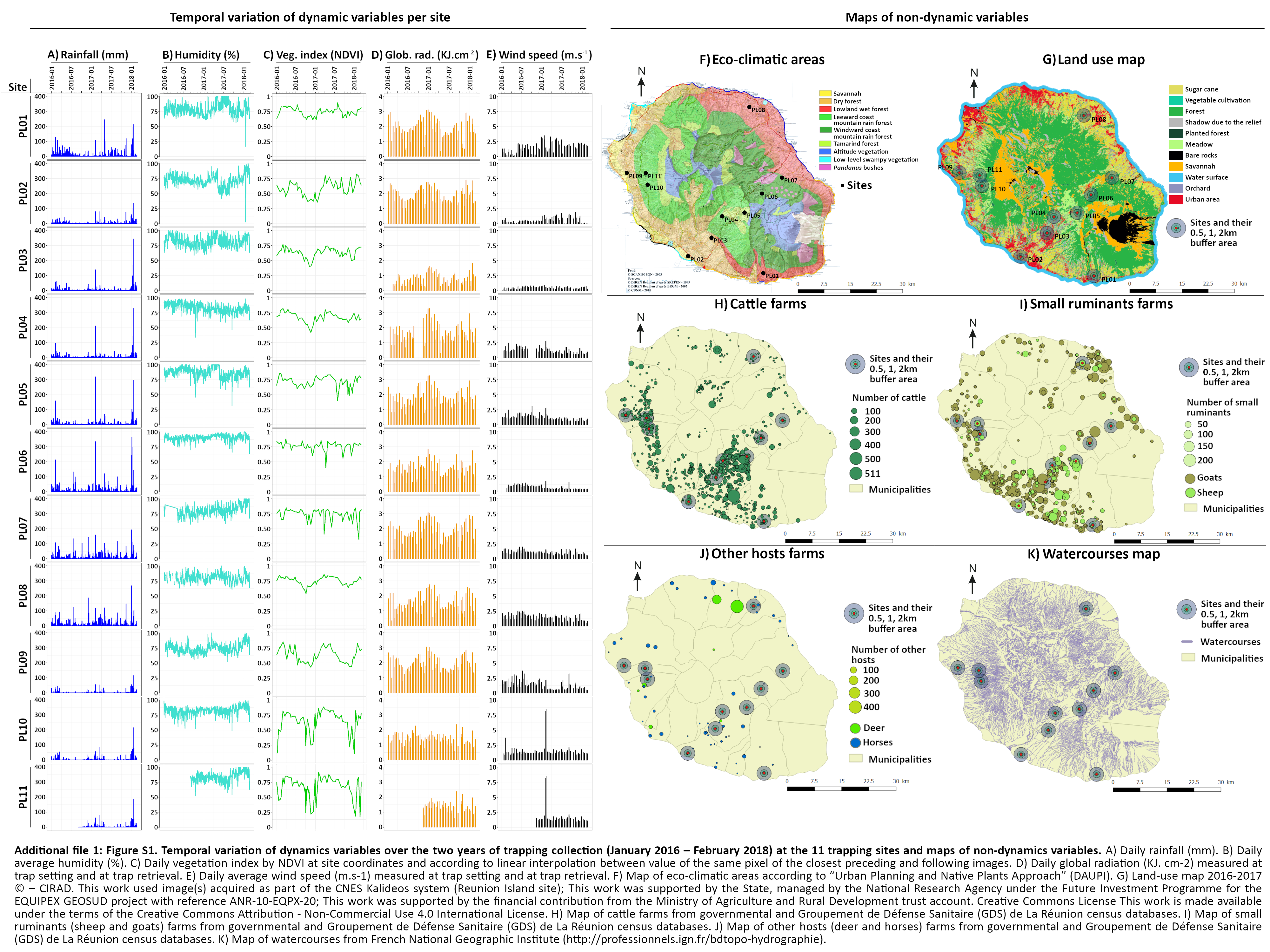

Supplement: Supplementary file 1 — Additional file 1: Figure S1. Temporal variation of dynamics variables over the two years of trapping collection (January 2016–February 2018) at the 11 trapping sites and maps of non-dynamics variables. [file 13071_2019_3812_MOESM1_ESM.png]

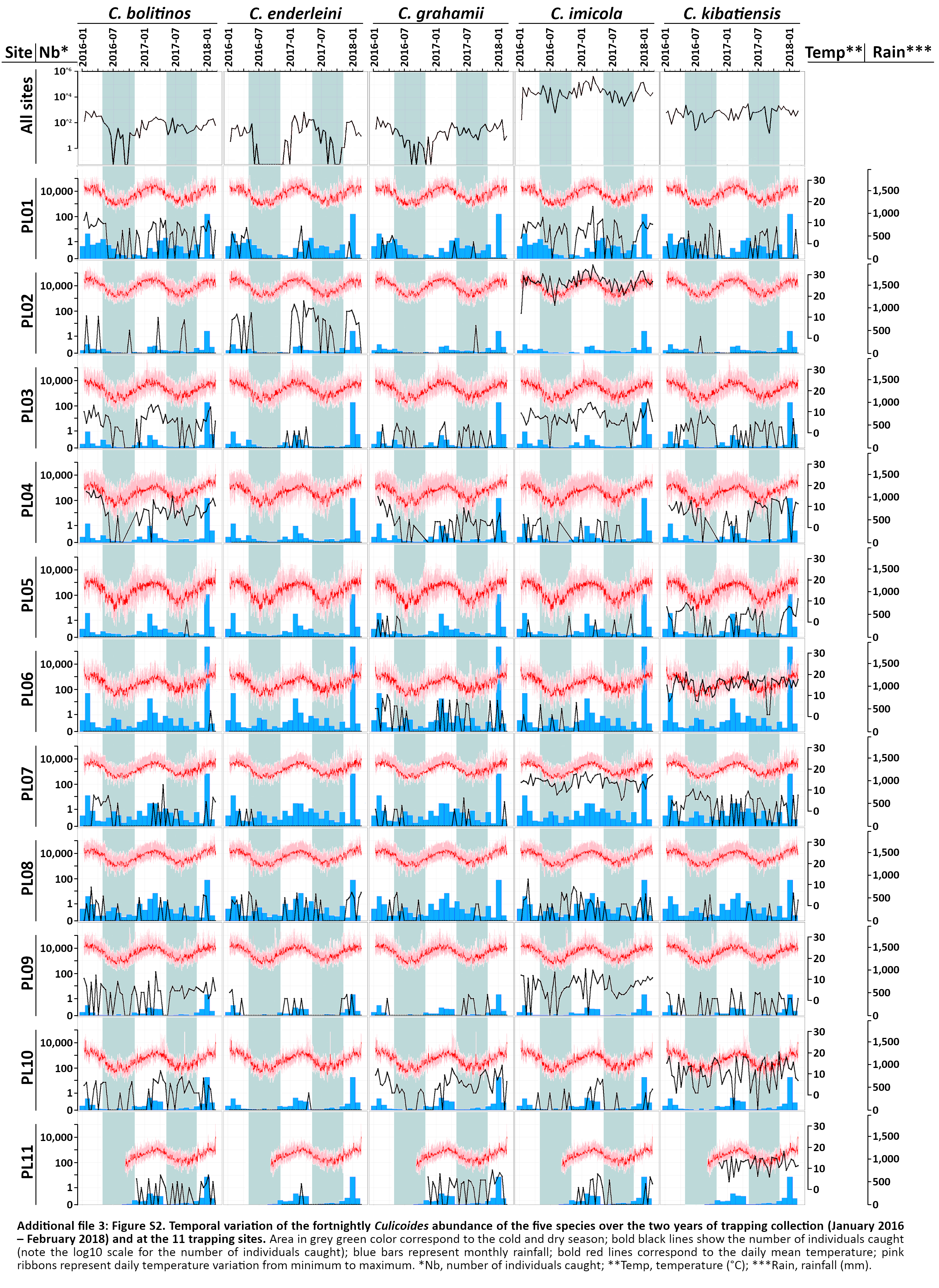

Supplement: Supplementary file 3 — Additional file 3: Figure S2. Temporal variation of the fortnightly Culicoides abundance of the five species over the two years of trapping collection (January 2016–February 2018) and at the 11 trapping sites. [file 13071_2019_3812_MOESM3_ESM.png]

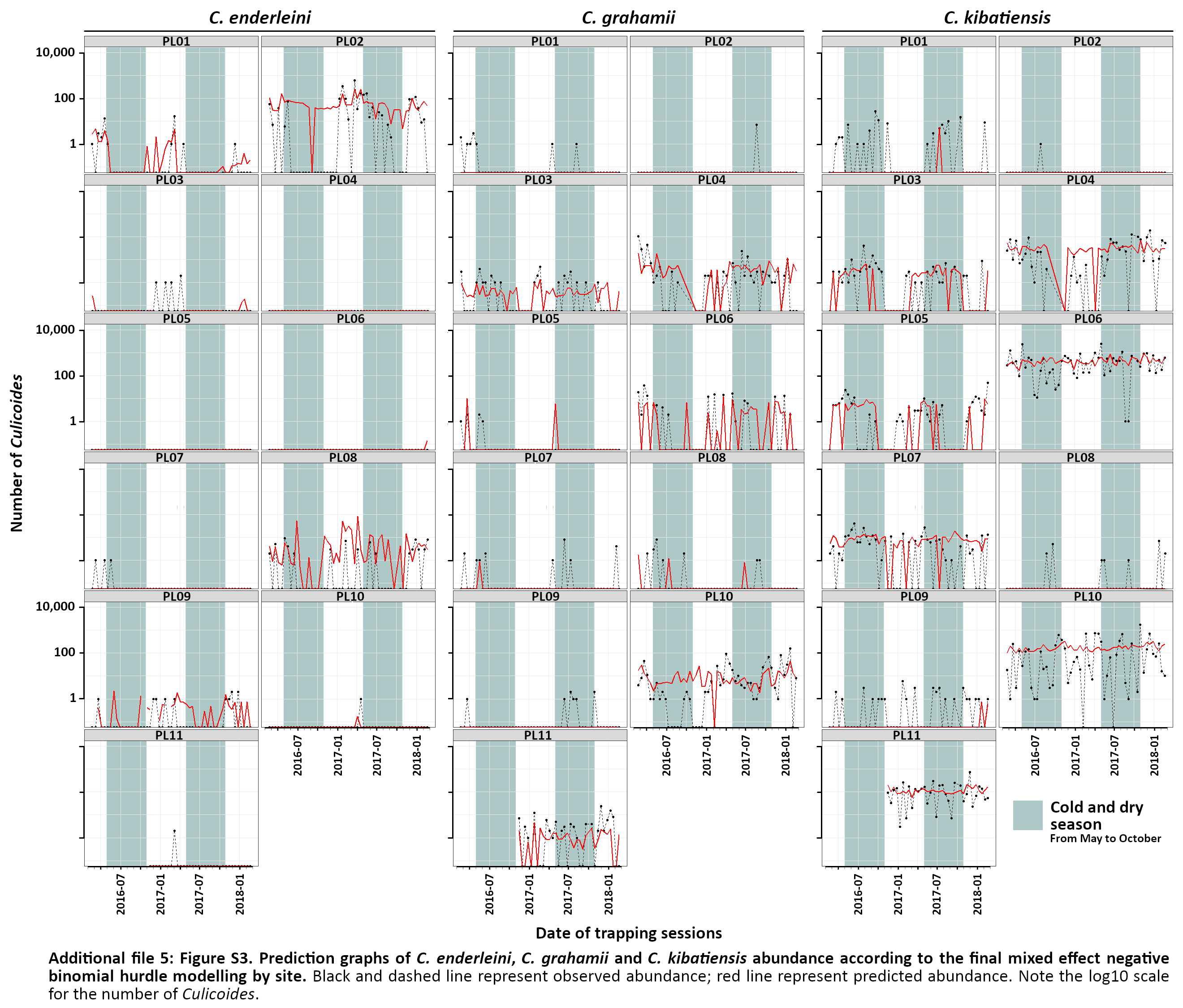

Supplement: Supplementary file 5 — Additional file 5: Figure S3. Prediction graphs of C. enderleini, C. grahamii and C. kibatiensis abundance according to the final mixed effect negative binomial hurdle modelling by site. [file 13071_2019_3812_MOESM5_ESM.png]
