# Supplementary material for: Modelling temporal dynamics of Culicoides Latreille (Diptera: Ceratopogonidae) populations on Reunion Island (Indian Ocean), vectors of viruses of veterinary importance
Source: Parasit Vectors. 2019 Nov 27;12:562. doi: 10.1186/s13071-019-3812-1 (PMC6880491; doi:10.1186/s13071-019-3812-1)
Supplement: Supplementary file 2 — Additional file 2: Table S1. Characteristics of trapping sites. Table S2. Temporal variables considered on mixed-effect negative binomial hurdle models. Table S3. Non-dynamic variables considered on mixed-effect negative binomial hurdle models. [file 13071_2019_3812_MOESM2_ESM.docx]

**Table S1: characteristics of trapping sites**

| Site number | Primary activity | Altitude (m) | Latitude | Longitude | Animals in the vicinity of traps | Number of animals in the vicinity of traps | Other host(s) present on the farm | Building opening (%) |
| --- | --- | --- | --- | --- | --- | --- | --- | --- |
| PL01 | Beef farm | 200 | -21.36 | 55.64 | Cattle | 50 | - | 25-75 |
| PL02 | Mixed farm | 40 | -21.32 | 55.44 | Cattle | 100 | Sheep, goats, pig, poultry | 75-100 |
| PL03 | Beef farm | 658 | -21.26 | 55.51 | Cattle | 38 | Poultry | 75-100 |
| PL04 | Beef farm | 1200 | -21.21 | 55.53 | Cattle | 50 | - | 25-75 |
| PL05 | Dairy farm | 1664 | -21.20 | 55.59 | Cattle | 57 | Sheep | 0-25 |
| PL06 | Dairy farm | 1223 | -21.16 | 55.63 | Cattle | 70 | - | 25-75 |
| PL07 | Beef farm | 557 | -21.11 | 55.69 | Cattle | 15 | - | Enclosure |
| PL08 | Mixed farm | 166 | -20.95 | 55.61 | Cattle | 9 | Sheep, goats, poultry | 0-25 |
| PL09 | Beef farm | 371 | -21.10 | 55.27 | Cattle | 14 | - | Enclosure |
| PL10 | Dairy farm | 1220 | -21.13 | 55.33 | Cattle | 50 | - | 0-25 |
| PL11 | Mixed farm | 1197 | -21.11 | 55.33 | Deer | 12 | Cattle, sheep, poultry, pig, donkey | Enclosure |

**Table S2: Temporal variables considered on NBH models, periods and location of measurements, transformations performed on them.**

| Categories of variables | Variables (data sources*) | Periods considered in relation to the catch | Transformations tested | CCM-defined lag | Measurement location | Nb meteo. station (mean distance**) | Units | Observed range |
| --- | --- | --- | --- | --- | --- | --- | --- | --- |
| Weather variables | Minimum temperature (a, b) | During catch, 2 prev. week | Qt3, qt4, qt5, log10 | Yes | Trap loc., at site coord. | 31 (3.53 km) | °C | [-2, 25.62] |
|  | Average temperature (a, b) | During catch | Qt3, qt4, qt5, log10 | Yes | Trap loc., at site coord. | 31 (3.53 km) | °C | [6.37, 28.57] |
|  | Maximum temperature (a, b) | During catch, 2 prev. week | Qt3, qt4, qt5, log10 | Yes | Trap loc., at site coord. | 31 (3.53 km) | °C | [13.49, 37.95] |
|  | Average humidity (a, b) | During catch | Qt3, qt4, qt5, log10 | Yes | Trap loc., nearest meteo. station | 14 (4.05 km) | % | [50, 100] |
|  | Daily rains (b) | Trap setting day, trap retrieval day | Qt3, qt4, qt5, log10 | Yes | Trap loc., at site coord. | 31 (3.53 km) | mm | [0, 146.3] |
|  | Average wind speed (b) | Trap setting day, trap retrieval day | Qt3, qt4, qt5, log10 | - | Nearest meteo. station | 25*** (3.27 km) | m.s^-1^ | [0, 8.5] |
|  | Daily global radiation (b) | Trap setting day, trap retrieval day | Qt3, qt4, qt5, log10 | - | Nearest meteo. station | 28 (3.27 km) | kJ.cm^-2^ | [0.16, 3.27] |
| Vegetation index | NDVI (c) | Trap setting day | Value * 100 | Yes | 250*250m pixel | - | No unit | [22, 88] |

*Data sources: a, thermo-hygro microchip recorders; b, Météo France and Cirad meteorological station; c, MODIS. ** Mean distance of the nearest meteorological stations (concerned sites for temperature and humidity: PL02, PL04, PL05, PL07, PL08, PL11). *** 10 of the 25 stations only provide wind at 10m above ground and concern sites PL04 to PL11. Abbreviations: QtX, splitting into quantiles of X classes; CCM, Cross Correlation Map; Nb, number; meteo., meteorological; loc., location.

**Table S3: Non-dynamic variables considered on NBH models.**

| Categories of variables | Variables (data sources*) | Measurement location | Units | Observed range |
| --- | --- | --- | --- | --- |
| Forested environment | Eco-climatic area (a) | At site coordinates | Categorical variable | x classes |
|  | % cover of each land use class (b) | At site coordinates | % | [0, 83.7] |
| Density of hosts | Number of target hosts (c) | At trap vicinity | nb individuals | [9, 100] |
|  | Farms density (d) | Buffer | nb/ buf. | [0, 13] - 0.5 km buf.  [2, 37] - 1 km buf.  [4, 69] - 2 km buf. |
|  | Density of total animals (d) | Buffer | nb/ buf. | [14, 343] - 0.5 km buf.  [22, 1130] - 1 km buf.  [22, 1859] - 2 km buf. |
|  | Density of cattle (d) | Buffer | nb/ buf. | [9, 263] - 0.5 km buf.  [16, 884] - 1 km buf.  [22, 1474] - 2 km buf. |
|  | Density of small ruminants (sheep and goats) (d) | Buffer | nb/ buf. | [0, 80] - 0.5 km buf.  [0, 246] - 1 km buf.  [0, 385] - 2 km buf. |
|  | Density of others animals (deer and horse) (d) | Buffer | nb/ buf. | [0, 12] - 0.5 km buf.  [0, 12] - 1 km buf.  [0, 52] - 2 km buf. |
| Water courses | Length of water courses (e) | Buffer | Km/ buf. | [0, 6.62] - 0.5 km buf.  [2.35, 27.88] - 1 km buf.  [8.02, 99.77] - 2 km buf. |
| Stable housing | Building opening size (c) | At trap vicinity | Categorical variable | x classes |

*Data sources: a, map of “Urban Planning and Native Plants Approach” (DAUPI); b, land-use map 2016-2017 © – CIRAD; c, recorded on the field; d, governmental and Groupement de Défense Sanitaire (GDS) de La Réunion census databases; e, French National Geographic Institute. Abbreviations: buf., buffer area.

© "Land use map 2016-2017 - CIRAD"; "This work used image(s) acquired as part of the CNES Kalideos system (Reunion Island site)"; "This work was supported by the State, managed by the National Research Agency under the Future Investment Programme for the EQUIPEX GEOSUD project with reference ANR-10-EQPX-20"; "This work was supported by the financial contribution from the Ministry of Agriculture "Agriculture and Rural Development" trust account. Creative Commons License This work is made available under the terms of the Creative Commons Attribution - Non-Commercial Use 4.0 International License.
