# Supplementary material for: Modelling temporal dynamics of Culicoides Latreille (Diptera: Ceratopogonidae) populations on Reunion Island (Indian Ocean), vectors of viruses of veterinary importance
Source: Parasit Vectors. 2019 Nov 27;12:562. doi: 10.1186/s13071-019-3812-1 (PMC6880491; doi:10.1186/s13071-019-3812-1)
Supplement: Supplementary file 4 — Additional file 4: Table S4. Final mixed-effect negative binomial hurdle model of risk factors associated with the count of C. bolitinos. Table S5. Final mixed-effect negative binomial hurdle model of risk factors associated with the count of C. enderleini. Table S6. Final mixed-effect negative binomial hurdle model of risk factors associated with the count of C. grahamii. Table S7. Final mixed-effect negative binomial hurdle model of risk factors associated with the count of C. imicola. Table S8. Final mixed-effect negative binomial hurdle model of risk factors associated with the count of C. kibatiensis. Table S9. Final mixed-effect negative binomial hurdle model of risk factors associated with the count of C. imicola without PL02 site. Table S10. Measurement of model fit (AIC), predictive accuracy or (AUC) and predictive performance (NRMSE) of the mixed effect negative binomial hurdle model for each species and each validation steps. [file 13071_2019_3812_MOESM4_ESM.docx]

**Table S4. Final mixed-effect negative binomial hurdle model of risk factors associated with the count of *C. bolitinos* (with farm random effects in the count part) ^a^.**

| **Variables (unit)** | **Classes** | **Coeff.** ^b^ | **SE** ^c^ | **OR or IRR** ^d^ | **95% CI** ^e^ | **Value of t-statistic** | **p value** |
| --- | --- | --- | --- | --- | --- | --- | --- |
| **Binary part (binomial with logit link)** | |  |  |  |  |  |  |
| ***Intercept*** | | -12.175 | 2.054 | - | - | -5.929 | <0.001 *** |
| Maximum temperature during the catch (°C) | | 0.120 | 0.056 | 1.13 | 1.01 - 1.26 | 2.131 | 0.033 * |
| Maximum temperature 14 days before catch (°C) | | 0.210 | 0.061 | 1.23 | 1.09 - 1.39 | 3.431 | <0.001 *** |
| Average rain from day 24 to day 21 before catch (mm) | | -0.067 | 0.024 | 0.94 | 0.89 - 0.98 | -2.816 | 0.005 ** |
| NDVI 18 days before catch | | 0.029 | 0.011 | 1.03 | 1.01 - 1.05 | 2.606 | 0.009 ** |
| % of land use by planted forest in 0.5 km radius (%) | | -3.513 | 1.860 | 0.03 | 0 - 1.14 | -1.889 | 0.059 . |
| Number of small ruminants in 0.5 km radius | | -0.044 | 0.006 | 0.96 | 0.95 - 0.97 | -7.117 | <0.001 *** |
| Number of farms in 2 km radius | | 0.075 | 0.014 | 1.08 | 1.05 - 1.11 | 5.510 | <0.001 *** |
| Minimum temperature between 2 trapping sessions (°C) | [-2, 7.04] | - | - | 1 | - |  | - |
|  | (7.04, 13.7] | 2.061 | 0.670 | 7.85 | 2.11 - 29.2 | 3.076 | 0.002 ** |
|  | (13.7, 16.9] | 1.026 | 0.711 | 2.79 | 0.69 - 11.24 | 1.443 | 0.149 |
|  | (16.9, 25.6] | 0.634 | 0.683 | 1.88 | 0.49 - 7.19 | 0.928 | 0.354 |
| **Count part (zero-truncated negative binomial with log link)** | | | | | | | |
| ***Intercept*** | | 1.096 | 1.848 | - | - | 0.593 | 0.553 |
| Log10 minimum temperature 10 days before the catch (°C) | | -4.654 | 1.165 | 0.010 | 0.00 – 0.09 | -3.994 | <0.001 *** |
| Maximum temperature 35 days before the catch (°C) | | 0.192 | 0.038 | 1.212 | 1.12 – 1.31 | 5.020 | <0.001 *** |
| Log10 rain during the day at trap retrieval (mm) | | 0.297 | 0.204 | 1.346 | 0.90 – 2.01 | 1.453 | 0.146 |
| Rain 9 days before catch (mm) | | 0.015 | 0.009 | 1.015 | 1.00 – 1.03 | 1.639 | 0.101 |
| Average log10 rain from day 25 to day 23 before catch (mm) | | 0.804 | 0.291 | 2.235 | 1.26 – 3.95 | 2.765 | 0.006 ** |
| % of land use by savannah in 0.5 km radius (%) | | -0.050 | 0.020 | 0.951 | 0.91 – 0.99 | -2.468 | 0.014 * |
| % of land use by planted forest in 1 km radius (%) | | -0.554 | 0.290 | 0.575 | 0.33 – 1.01 | -1.911 | 0.056 . |
| Number of cattle in 1 km radius | | 0.003 | 0.002 | 1.003 | 1.00 – 1.01 | 1.451 | 0.147 |
| Rain during the day at trap setting (mm) | [0, 0.2] | - | - | 1 | - |  | - |
|  | (0.2, 1.5] | -0.239 | 0.272 | 0.787 | 0.46 – 1.34 | -0.881 | 0.378 |
|  | (1.5, 6.92] | 0.109 | 0.289 | 1.115 | 0.63 – 1.96 | 0.377 | 0.706 |
|  | (6.92, 146] | 0.636 | 0.298 | 1.890 | 1.05 – 3.39 | 2.135 | 0.033 * |
| Global radiation during the day at trap setting (kJ.cm^-2^) | [0.3, 1.09] | - | - | 1 | - |  | - |
|  | (1.09, 1.53] | 0.415 | 0.300 | 1.515 | 0.84 – 2.73 | 1.385 | 0.166 |
|  | (1.53, 1.97] | 0.669 | 0.326 | 1.953 | 1.03 – 3.70 | 2.056 | 0.040 * |
|  | (1.97, 3.27] | -0.057 | 0.386 | 0.945 | 0.44 – 2.01 | -0.147 | 0.883 |
| Global radiation during the day at trap retrieval (kJ.cm^-2^) | [0.16, 1.25] | - | - | 1 | - |  | - |
|  | (1.25, 1.8] | 0.527 | 0.271 | 1.695 | 1.00 – 2.88 | 1.944 | 0.052 . |
|  | (1.8, 3.25] | 0.851 | 0.377 | 2.343 | 1.12 – 4.91 | 2.257 | 0.024 * |
| Building opening size (%) | [0, 25] | - | - | 1 | - |  | - |
|  | (25, 75] | 1.403 | 0.341 | 4.069 | 2.08 – 7.94 | 4.112 | <0.001 *** |
|  | (75, 100] | 1.341 | 0.400 | 3.823 | 1.75 – 8.37 | 3.353 | <0.001 *** |
|  | Enclosure | 0.766 | 0.323 | 2.151 | 1.14 – 4.05 | 2.374 | 0.018 * |

^a^ Variance component values (standard deviation) was 1.226e-4 for farm in the count part. ^b^ Coeff., value of the regression coefficient. ^c^ SE, standard error. ^d^ OR, adjusted odds ratio; IRR, incidence rate ratio. In the column, ORs are shown for the binary part and IRRs are shown for the count part. ^e^ CI, confidence interval of adjusted ORs or IRRs.

**Table S5: Final mixed-effect negative binomial hurdle model of risk factors associated with the count of *C. enderleini* (with farm random effects in the count part) ^a^.**

| **Variables (unit)** | **Classes** | **Coeff.** ^b^ | **SE** ^c^ | **OR or IRR** ^d^ | **95% CI** ^e^ | **Value of t-statistic** | **p value** |
| --- | --- | --- | --- | --- | --- | --- | --- |
| **Binary part (binomial with logit link)** | |  |  |  |  |  |  |
| ***Intercept*** | | -3.666 | 6.416 | - | - | -0.677 | 0.498 |
| Maximum temperature during the catch (°C) | | 0.246 | 0.087 | 1.28 | 1.08 – 1.52 | 2.833 | 0.005 ** |
| Maximum temperature between 2 trapping sessions (°C) | | 0.338 | 0.120 | 1.4 | 1.11 – 1.77 | 2.812 | 0.005 ** |
| Average humidity from day 27 to day 17 before catch (%) | | -0.080 | 0.044 | 0.92 | 0.85 – 1.01 | -1.805 | 0.071 . |
| Average humidity from day 41 to day 35 before catch (%) | | -0.126 | 0.05 | 0.88 | 0.8 – 0.97 | -2.513 | 0.012 * |
| Average rain from day 48 to day 44 before catch (mm) | | 0.062 | 0.034 | 1.06 | 1 – 1.14 | 1.830 | 0.067 . |
| Average wind during the day at trap setting (m.s^-1^) | | -1.209 | 0.395 | 0.3 | 0.14 – 0.65 | -3.064 | 0.002 ** |
| NDVI | | 0.051 | 0.023 | 1.05 | 1.01 – 1.1 | 2.174 | 0.030 * |
| % land use by forest in 2 km radius (%) | | -0.045 | 0.022 | 0.96 | 0.92 – 1 | -2.031 | 0.042 * |
| % land use by orchard in 2 km radius (%) | | -0.088 | 0.062 | 0.92 | 0.81 – 1.03 | -1.420 | 0.156 |
| Rain during the day at trap retrieval (mm) | (0, 0.1] | - | - | 1 | - |  | - |
|  | [0.1, 1.1] | 1.164 | 0.632 | 3.2 | 0.93 – 11.06 | 1.842 | 0.065 . |
|  | [1.1, 6.3] | -0.415 | 0.721 | 0.66 | 0.16 – 2.71 | -0.576 | 0.564 |
|  | [6.3, 117.7] | -0.740 | 0.679 | 0.48 | 0.13 – 1.8 | -1.090 | 0.276 |
| **Count part (zero-truncated negative binomial with log link)** | | | | | | | |
| ***Intercept*** | | -10.487 | 3.756 | - | - | -2.792 | 0.005 ** |
| Log10 minimum temperature between 2 trapping sessions (°C) | | 4.719 | 3.046 | 112.05 | 0.29 – 4.4e5 | 1.549 | 0.121 |
| Log10 rain 9 days before catch (mm) | | 1.126 | 0.344 | 3.084 | 1.57 – 6.06 | 3.272 | 0.001 ** |
| Number of small ruminants in 1 km radius | | 0.070 | 0.016 | 1.073 | 1.04 – 1.11 | 4.514 | <0.001 *** |
| Number of total animals in 1 km radius | | 0.022 | 0.014 | 1.022 | 0.99 – 1.05 | 1.521 | 0.128 |
| Average wind during the day at trap retrieval (m.s^-1^) | (0, 0.512] | - | - | 1 | - |  | - |
|  | [0.512, 0.9] | -0.491 | 0.549 | 0.612 | 0.21 – 1.80 | -0.893 | 0.372 |
|  | [0.9, 1.2] | -0.281 | 0.760 | 0.755 | 0.17 – 3.35 | -0.370 | 0.711 |
|  | [1.2, 1.46] | 1.713 | 0.588 | 5.545 | 1.75 – 17.54 | 2.915 | 0.004 ** |
|  | [1.46, 8.5) | -1.982 | 0.916 | 0.138 | 0.02 – 0.83 | -2.164 | 0.030 * |
| Eco-climatic area | Df & sav. ^f^ | - | - | 1 | - |  | - |
|  | Lwf ^g^ | 1.157 | 1.059 | 3.182 | 0.4 – 25.34 | 1.093 | 0.274 |
|  | Mrf & Tf ^h^ | -3.401 | 2.666 | 0.033 | 0 – 6.19 | -1.276 | 0.202 |

^a^ Variance component values (standard deviation) was 2.837e-5 for farm in the count part. ^b^ Coeff., value of the regression coefficient. ^c^ SE, standard error. ^d^ OR, adjusted odds ratio; IRR, incidence rate ratio. In the column, ORs are shown for the binary part and IRRs are shown for the count part. ^e^ CI, confidence interval of adjusted ORs or IRRs. ^f^ Dry forest and savannah. ^g^ Lowland wet forest. ^h^ Mountain rain forest & tamarind forest.

**Table S6: Final mixed-effect negative binomial hurdle model of risk factors associated with the count of *C. grahamii* (with farm random effects in the count part) ^a^.**

| **Variables (unit)** | **Classes** | **Coeff.** ^b^ | **SE** ^c^ | **OR or IRR** ^d^ | **95% CI** ^e^ | **Value of t-statistic** | **p value** |
| --- | --- | --- | --- | --- | --- | --- | --- |
| **Binary part (binomial with logit link)** | |  |  |  |  |  |  |
| ***Intercept*** | | -15.818 | 3.081 | - | - | -5.134 | <0.001 *** |
| Average humidity from day 20 to day 15 before catch (%) | | 0.049 | 0.027 | 1.05 | 1 – 1.11 | 1.816 | 0.071 . |
| Average humidity from day 48 to day 43 before catch (%) | | 0.064 | 0.028 | 1.07 | 1.01 – 1.13 | 2.341 | 0.019 * |
| Log10 Average rain from day 3 to day 2 before catch (mm) | | -0.723 | 0.442 | 0.49 | 0.2 – 1.15 | -1.637 | 0.102 |
| Average rain from day 29 to day 24 before catch (mm) | | -0.042 | 0.021 | 0.96 | 0.92 - 1 | -1.969 | 0.049 * |
| Rain 48 days before catch (mm) | | 0.036 | 0.018 | 1.04 | 1 – 1.07 | 2.028 | 0.043 * |
| Average wind during the day at trap setting (m.s^-1^) | | -0.599 | 0.337 | 0.55 | 0.28 – 1.06 | -1.774 | 0.076 . |
| NDVI | | 0.022 | 0.015 | 1.02 | 0.99 – 1.05 | 1.504 | 0.133 |
| NDVI 31 days before catch | | 0.043 | 0.016 | 1.04 | 1.01 – 1.08 | 2.768 | 0.006 ** |
| Number of deer and horses in 2 km radius | | 0.034 | 0.011 | 1.03 | 1.01 – 1.06 | 3.095 | 0.002 ** |
| Minimum temperature during the catch (°C) | [-2, 12.1] | - | - | 1 | - |  | - |
|  | (12.1, 15.1] | 1.213 | 0.458 | 3.36 | 1.37 – 8.25 | 2.651 | 0.008 ** |
|  | (15.1, 18] | 0.370 | 0.513 | 1.45 | 0.53 – 3.96 | 0.722 | 0.470 |
|  | (18, 21] | 0.624 | 0.554 | 1.87 | 0.63 – 5.52 | 1.126 | 0.260 |
|  | (21, 25.6] | 0.411 | 0.797 | 1.51 | 0.32 – 7.19 | 0.517 | 0.605 |
| Eco-climatic area | Df, sav. & Tf ^f^ | - | - | 1 | - |  | - |
|  | Lwf ^g^ | 0.447 | 0.667 | 1.56 | 0.42 – 5.78 | 0.670 | 0.503 |
|  | Wcmrf ^h^ | 0.311 | 0.575 | 1.36 | 0.44 – 4.21 | 0.540 | 0.589 |
|  | Lcmrf ^i^ | 2.648 | 0.517 | 14.12 | 5.13 – 38.88 | 5.124 | <0.001 *** |
| **Count part (zero-truncated negative binomial with log link)** | | | | | | | |
| ***Intercept*** | | 1.765 | 1.235 |  |  | 1.430 | 0.153 |
| NDVI 21 days before catch | | -0.020 | 0.011 | 0.950 | 0.96 – 1 | -1.887 | 0.059 . |
| Rain during the day at trap setting (mm) | [0, 0.2] | - | - | 1 | - |  | - |
|  | (0.2, 1.5] | 0.936 | 0.455 | 2.549 | 1.05 – 6.21 | 2.059 | 0.040 * |
|  | (1.5, 6.92] | 1.489 | 0.516 | 4.435 | 1.61 – 12.20 | 2.884 | 0.004 ** |
|  | (6.92, 146] | 1.472 | 0.499 | 4.360 | 1.64 – 11.60 | 2.949 | 0.003 ** |
| Rain during the day at trap retrieval (mm) | [0, 0.1] | - | - | 1 | - |  | - |
|  | (0.1, 1.1] | -0.864 | 0.463 | 0.421 | 0.17 – 1.04 | -1.868 | 0.062 . |
|  | (1.1, 6.3] | -1.899 | 0.469 | 0.150 | 0.06 – 0.38 | -4.052 | <0.001 *** |
|  | (6.3, 117.7] | -0.528 | 0.457 | 0.590 | 0.24 – 1.44 | -1.156 | 0.248 |
| Global radiation during the day at trap setting (kJ.cm^-2^) | [0.3, 1.01] | - | - | 1 | - |  | - |
|  | (1.01, 1.38] | -0.333 | 0.442 | 0.717 | 0.30 – 1.70 | -0.753 | 0.451 |
|  | (1.38, 1.68] | -0.134 | 0.491 | 0.875 | 0.33 – 2.29 | -0.272 | 0.786 |
|  | (1.68, 2.1] | 1.249 | 0.556 | 3.486 | 1.17 – 10.37 | 2.245 | 0.025 * |
|  | (2.1, 3.27] | -1.267 | 0.731 | 0.282 | 0.07 – 1.18 | -1.733 | 0.083 . |
| Eco-climatic area | Df, sav. & Tf | - | - | 1 | - |  | - |
|  | Lwf | -0.301 | 0.948 | 0.740 | 0.12 – 4.74 | -0.318 | 0.751 |
|  | Wcmrf | 1.990 | 0.943 | 7.316 | 1.15 – 46.43 | 2.111 | 0.035 * |
|  | Lcmrf | 1.340 | 0.769 | 3.818 | 0.85 – 17.23 | 1.743 | 0.081 . |
| Building opening size (%) | [0, 25] | - | - | 1 | - |  | - |
|  | (25, 75] | -0.696 | 0.467 | 0.499 | 0.20 – 1.25 | -1.490 | 0.136 |
|  | (75, 100] | -2.009 | 0.547 | 0.134 | 0.05 – 0.39 | -3.676 | <0.001 *** |
|  | Enclosure | -0.732 | 0.446 | 0.481 | 0.20 – 1.15 | -1.642 | 0.101 |

^a^ variance component values (standard deviation) was 4.884e-5 for farm in the count part. ^b^ Coeff., value of the regression coefficient. ^c^ SE, standard error. ^d^ OR, adjusted odds ratio; IRR, incidence rate ratio. In the column, ORs are shown for the binary part and IRRs are shown for the count part. ^e^ CI, confidence interval of adjusted ORs or IRRs. ^f^ Df, sav. & Ft, dry forest, savannah & tamarind forest. ^g^ Lwf, lowland wet forest. ^h^ Wcmrf, windward coast mountain rain forest. ^i^ Lcmrf, leeward coast mountain rain forest.

**Table S7: Final mixed-effect negative binomial hurdle model of risk factors associated with the count of *C. imicola* (with farm random effects in the count part) ^a^.**

| **Variables (unit)** | **Classes** | **Coeff.** ^b^ | **SE** ^c^ | **OR or IRR** ^d^ | **95% CI** ^e^ | **Value of t-statistic** | **p value** |
| --- | --- | --- | --- | --- | --- | --- | --- |
| **Binary part (binomial with logit link)** | |  |  |  |  |  |  |
| ***Intercept*** | | -9.855 | 2.157 | - | - | -4.568 | <0.001 *** |
| Maximum temperature during the catch (°C) | | 0.160 | 0.058 | 1.17 | 1.05 – 1.31 | 2.772 | 0.006 ** |
| Maximum temperature between 2 trap sessions (°C) | | 0.214 | 0.079 | 1.24 | 1.06 – 1.45 | 2.697 | 0.007 ** |
| Rain during the day at trap retrieval (mm) | | 0.028 | 0.013 | 1.03 | 1 – 1.05 | 2.175 | 0.030 * |
| Rain 4 days before catch (mm) | | -0.033 | 0.015 | 0.97 | 0.94 - 1 | -2.263 | 0.024 * |
| Average wind during the day at trap setting (m.s^-1^) | | -0.477 | 0.297 | 0.62 | 0.35 – 1.11 | -1.605 | 0.109 |
| % land use by urban area in 2 km radius (%) | | 0.052 | 0.026 | 1.05 | 1 – 1.11 | 2.013 | 0.044 * |
| Number of small ruminants in 1 km radius | | -0.010 | 0.004 | 0.99 | 0.98 – 0.999 | -2.261 | 0.024 * |
| Building opening size (%) | [0, 25] | - | - | 1 | - |  | - |
|  | (25, 75] | -0.020 | 0.441 | 0.98 | 0.41 – 2.33 | -0.045 | 0.964 |
|  | (75, 100] & enclosure | 2.746 | 0.499 | 15.58 | 5.86 – 41.39 | 5.506 | <0.001 *** |
| **Count part (zero-truncated negative binomial with log link)** | | | | | | | |
| ***Intercept*** | | -2.718 | 1.755 | - | - | -1.549 | 0.121 |
| Maximum temperature during the catch (°C) | | 0.190 | 0.028 | 1.209 | 1.14 – 1.28 | 6.671 | <0.001 *** |
| Maximum temperature between 2 trapping sessions (°C) | | 0.090 | 0.038 | 1.094 | 1.02 – 1.18 | 2.352 | 0.019 * |
| Humidity at day 26 before catch (%) | | -0.027 | 0.011 | 0.974 | 0.95 – 1.00 | -2.360 | 0.018 * |
| Rain during the day at trap retrieval (mm) | | -0.013 | 0.006 | 0.987 | 0.97 – 1.00 | -2.012 | 0.044 * |
| NDVI at day 50 before catch | | -0.020 | 0.009 | 0.980 | 0.96 – 1.00 | -2.237 | 0.025 * |
| % land use by planted forest in 1 km radius (%) | | -0.735 | 0.146 | 0.480 | 0.36 – 0.64 | -5.028 | <0.001 *** |
| % land use by bare rock in 2 km radius (%) | | 4.195 | 0.179 | 66.35 | 46.7 – 94.3 | 23.397 | <0.001 *** |
| Number of deer and horses in 2 km radius | | -0.031 | 0.012 | 0.970 | 0.95 – 0.99 | -2.493 | 0.013 * |
| Rain during the day at trap setting (mm) | [0, 0.2] | - | - | 1 | - |  | - |
|  | (0.2, 1.5] | -0.815 | 0.276 | 0.443 | 0.26 – 0.76 | -2.959 | 0.003 ** |
|  | (1.5, 6.92] | 0.037 | 0.261 | 1.038 | 0.62 – 1.73 | 0.143 | 0.886 |
|  | (6.92, 146] | -0.332 | 0.237 | 0.718 | 0.45 – 1.14 | -1.399 | 0.162 |
| NDVI | [22, 60] | - | - | 1 | - |  | - |
|  | (60, 67] | 0.440 | 0.251 | 1.552 | 0.95 – 2.54 | 1.753 | 0.080 . |
|  | (67, 73] | 0.165 | 0.275 | 1.180 | 0.69 – 2.02 | 0.601 | 0.548 |
|  | (73, 78] | -0.441 | 0.278 | 0.643 | 0.37 – 1.11 | -1.587 | 0.112 |
|  | (78, 88] | 0.527 | 0.306 | 1.694 | 0.93 – 3.08 | 1.724 | 0.085 . |
| Eco-climatic area | Lwf ^f^ | - | - | 1 | - |  | - |
|  | Df & sav. ^g^ | 1.524 | 0.286 | 4.590 | 2.62 – 8.05 | 5.322 | <0.001 *** |
|  | Wcmrf ^h^ | 4.621 | 0.309 | 101.6 | 55.4 – 186.4 | 14.937 | <0.001 *** |
|  | Lcmrf & Tf ^i^ | 1.368 | 0.294 | 3.929 | 2.21 – 6.99 | 4.653 | <0.001 *** |

^a^ variance component values (standard deviation) was 3.316e-05for farm in the count part. ^b^ Coeff., value of the regression coefficient. ^c^ SE, standard error. ^d^ OR, adjusted odds ratio; IRR, incidence rate ratio. In the column, ORs are shown for the binary part and IRRs are shown for the count part. ^e^ CI, confidence interval of adjusted ORs or IRRs. ^f^ Lwf, lowland wet forest. ^g^ Df & sav., dry forest & savannah. ^h^ Wcmrf, windward coast mountain rain forest. ^i^ Lcmrf & Tf, leeward coast mountain rain forest & tamarind forest.

**Table S8: Final mixed-effect negative binomial hurdle model of risk factors associated with the count of *C. kibatiensis* (with farm random effects in the count part) ^a^.**

| **Variables (unit)** | **Classes** | **Coeff.** ^b^ | **SE** ^c^ | **OR or IRR** ^d^ | **95% CI** ^e^ | **Value of t-statistic** | **p value** |
| --- | --- | --- | --- | --- | --- | --- | --- |
| **Binary part (binomial with logit link)** | |  |  |  |  |  |  |
| ***Intercept*** | | 6.984 | 3.846 | - | - | 1.816 | 0.069 . |
| Maximum temperature during the catch (°c) | | -0.095 | 0.048 | 0.91 | 0.83 - 1 | -1.990 | 0.047 * |
| Maximum temperature between 2 trapping sessions (°C) | | -0.369 | 0.086 | 0.69 | 0.58 – 0.82 | -4.276 | <0.001 *** |
| Average humidity from day 50 to day 3 before catch (%) | | 0.053 | 0.032 | 1.05 | 0.99 – 1.12 | 1.635 | 0.102 |
| Log10 average rain from day 11 to day 10 before catch (mm) | | 0.747 | 0.402 | 2.11 | 0.96 – 4.64 | 1.857 | 0.063 . |
| NDVI 29 days before catch | | 0.038 | 0.017 | 1.04 | 1.01 – 1.07 | 2.293 | 0.022 * |
| % land use by bare rock in 2 km radius (%) | | -1.504 | 0.539 | 0.22 | 0.08 – 0.64 | -2.789 | 0.005 ** |
| Number of deer and horses in 2 km radius | | 0.038 | 0.019 | 1.04 | 1 – 1.08 | 2.006 | 0.045 * |
| Eco-climatic area | Df & sav. ^f^ | - | - | 1 | - |  | - |
|  | Lwf ^g^ | -1.958 | 0.592 | 0.14 | 0.04 – 0.45 | -3.321 | <0.001 *** |
|  | Wcmrf ^h^ | -0.907 | 0.674 | 0.4 | 0.11 – 1.51 | -1.346 | 0.178 |
|  | Lcmrf ^i^ | -0.544 | 0.588 | 0.58 | 0.18 – 1.84 | -0.924 | 0.355 |
|  | Tf ^j^ | -2.465 | 0.783 | 0.09 | 0.02 – 0.39 | -3.148 | 0.002 ** |
| **Count part (zero-truncated negative binomial with log link)** | | | | | | | |
| ***Intercept*** | | 3.305 | 1.601 | - | - | 2.064 | 0.039 * |
| Minimum temperature between 2 trap sessions (°C) | | -0.093 | 0.054 | 0.911 | 0.82 – 1.01 | -1.731 | 0.083 . |
| Maximum temperature 44 days before catch (°C) | | -0.077 | 0.036 | 0.926 | 0.86 – 0.99 | -2.147 | 0.032 * |
| Global radiation during the day at trap retrieval (kJ.cm^-2^) | | -0.304 | 0.187 | 0.738 | 0.51 – 1.06 | -1.626 | 0.104 |
| Number of deer and horses in 0.5 km radius | | 0.141 | 0.065 | 1.151 | 1.01 – 1.31 | 2.166 | 0.030 * |
| Number of deer and horses in 2 km radius | | 0.032 | 0.015 | 1.033 | 1.00 – 1.06 | 2.116 | 0.034 * |
| Minimum temperature during the catch (°C) | [-2, 12.1] | - | - | 1 | - |  | - |
|  | (12.1, 15.1] | 0.454 | 0.251 | 1.574 | 0.96 – 2.57 | 1.811 | 0.070 . |
|  | (15.1, 18] | -0.070 | 0.333 | 0.932 | 0.49 – 1.79 | -0.210 | 0.834 |
|  | (18, 21] | -0.427 | 0.458 | 0.652 | 0.27 – 1.60 | -0.933 | 0.351 |
|  | (21, 25.6] | 0.780 | 0.727 | 2.182 | 0.52 – 9.08 | 1.073 | 0.283 |
| Rain during the day at trap setting (mm) | [0, 0.2] | - | - | 1 | - |  | - |
|  | (0.2, 1.5] | 0.5995 | 0.264 | 1.821 | 1.09 – 3.05 | 2.272 | 0.023 * |
|  | (1.5, 6.92] | 0.987 | 0.294 | 2.684 | 1.51 – 4.78 | 3.357 | <0.001 *** |
|  | (6.92, 146] | 0.706 | 0.313 | 2.025 | 1.10 – 3.74 | 2.258 | 0.024 * |
| Eco-climatic area | Df & sav. | - | - | 1 | - |  | - |
|  | Lwf | 1.610 | 0.93 | 5.002 | 0.81 – 30.96 | 1.731 | 0.083 . |
|  | Wcmrf | 3.182 | 1.027 | 24.103 | 3.22 – 180.4 | 3.099 | 0.002 ** |
|  | Lcmrf | 1.691 | 1.055 | 5.425 | 0.69 – 42.91 | 1.602 | 0.109 |
|  | Tf | -0.125 | 1.422 | 0.883 | 0.05 – 14.34 | -0.088 | 0.930 |

^a^ variance component values (standard deviation) was 0.5962 for farm in the count part. ^b^ Coeff., value of the regression coefficient. ^c^ SE, standard error. ^d^ OR, adjusted odds ratio; IRR, incidence rate ratio. In the column, ORs are shown for the binary part and IRRs are shown for the count part. ^e^ CI, confidence interval of adjusted ORs or IRRs. ^f^ Df & sav., dry forest & savannah. ^g^ Lwf, lowland wet forest. ^h^ Wcmrf, windward coast mountain rain forest. ^i^ Lcmrf, leeward coast mountain rain forest. ^j^ Tf, tamarind forest.

**Table S9: Final mixed-effect negative binomial hurdle model of risk factors associated with the count of *C. imicola* without PL02 site (with farm random effects in the count part) ^a^.**

| **Variables (unit)** | **Classes** | **Coeff.** ^b^ | **SE** ^c^ | **OR or IRR** ^d^ | **95% CI** ^e^ | **Value of t-statistic** | **p value** |
| --- | --- | --- | --- | --- | --- | --- | --- |
| **Binary part (binomial with logit link)** | | | | | | | |
| ***Intercept*** | | -11.385 | 2.364 | - | - | -4.816 | <0.001 *** |
| Maximum temperature during the catch (°C) | | 0.164 | 0.059 | 1.18 | 1.05 – 1.32 | 2.768 | 0.006 ** |
| Maximum temperature between 2 trapping sessions (°C) | | 0.181 | 0.084 | 1.2 | 1.02 – 1.41 | 2.158 | 0.031 * |
| Rain during the day at trap retrieval (mm) | | 0.025 | 0.012 | 1.02 | 1 – 1.05 | 2.039 | 0.041 * |
| Rain 4 days before catch (mm) | | -0.036 | 0.016 | 0.96 | 0.94 – 0.99 | -2.302 | 0.021 * |
| Log10 average rain from day 9 to day 8 before catch (mm) | | 0.882 | 0.418 | 2.42 | 1.07– 5.48 | 2.111 | 0.035 * |
| NDVI | | 0.023 | 0.015 | 1.02 | 0.99 – 1.05 | 1.541 | 0.123 |
| % land use by urban area in 2 km radius (%) | | 0.064 | 0.031 | 1.07 | 1 – 1.13 | 2.077 | 0.038 * |
| Number of small ruminants in 0.5 km radius | | -0.027 | 0.009 | 0.97 | 0.96 – 0.99 | -2.860 | 0.004 ** |
| Building opening size (%) | [0, 25] | - | - | 1 | - |  | - |
|  | (25, 75] | -0.516 | 0.522 | 0.6 | 0.21 – 1.66 | -0.988 | 0.323 |
|  | (75, 100] & enclosure | 2.916 | 0.535 | 18.47 | 6.47 – 52.7 | 5.452 | <0.001 *** |
| **Count part (zero-truncated negative binomial with log link)** | | | | | | | |
| ***Intercept*** | | 1.306 | 1.379 | - | - | 0.947 | 0.344 |
| Maximum temperature during the catch (°C) | | 0.125 | 0.040 | 1.133 | 1.05 – 1.23 | 3.091 | 0.002 ** |
| Maximum temperature 13 days before catch (°C) | | 0.080 | 0.045 | 1.083 | 0.99 – 1.18 | 1.791 | 0.073 . |
| Humidity 6 days before catch (%) | | -0.026 | 0.012 | 0.975 | 0.95 – 1 | -2.162 | 0.031 * |
| Log10 average rain from day 18 to day 17 before catch (mm) | | 0.460 | 0.269 | 1.584 | 0.94 – 2.68 | 1.710 | 0.087 . |
| Number of animals at the vicinity of the trap | | -0.030 | 0.008 | 0.970 | 0.95 – 0.99 | -3.585 | <0.001 *** |
| Number of small ruminants in 1 km radius | | -0.050 | 0.010 | 0.951 | 0.93 – 0.97 | -4.825 | <0.001 *** |
| Number of deer and horses in 2 km radius | | -0.064 | 0.014 | 0.938 | 0.91 – 0.96 | -4.523 | <0.001 *** |
| Rain during the day at trap setting (mm) | [0, 0.3] | - | - | 1 | - |  | - |
|  | (0.3, 1.72] | -0.805 | 0.292 | 0.447 | 0.25 – 0.79 | -2.754 | 0.006 ** |
|  | (1.72, 6.92] | 0.386 | 0.285 | 1.471 | 0.84 – 2.57 | 1.356 | 0.175 |
|  | (6.92, 146] | -0.271 | 0.277 | 0.763 | 0.44 – 1.31 | -0.978 | 0.328 |
| Eco-climatic area | Df & sav. ^f^ | - | - | 1 | - |  | - |
|  | Lwf ^g^ | 0.095 | 0.362 | 1.100 | 0.54 – 2.23 | 0.264 | 0.792 |
|  | Wcmrf ^h^ | 1.278 | 0.409 | 3.591 | 1.61 – 8.01 | 3.126 | 0.002 ** |
|  | Lcmrf & Tf ^i^ | 0.584 | 0.351 | 1.794 | 0.90 – 3.57 | 1.666 | 0.096 . |

^a^ variance component values (standard deviation) was 5.028e-05 for farm in the count part. ^b^ Coeff., value of the regression coefficient. ^c^ SE, standard error. ^d^ OR, adjusted odds ratio; IRR, incidence rate ratio. In the column, ORs are shown for the binary part and IRRs are shown for the count part. ^e^ CI, confidence interval of adjusted ORs or IRRs. ^f^ Df & sav., dry forest & savannah. ^g^ Lwf, lowland wet forest. ^h^ Wcmrf, windward coast mountain rain forest. ^i^ Lcmrf & Tf, leeward coast mountain rain forest & tamarind forest.

**Table S10: measurement of model fit (AIC), predictive accuracy or (AUC) and predictive performance (NRMSE) of the mixed effect NBH model for each species and each validation steps.**

|  |  | Training model | | Internal validation LOOCV | | External validation | |
| --- | --- | --- | --- | --- | --- | --- | --- |
|  |  | AIC | AUC or NRMSE (%) | AUC or NRMSE (%) | Variation | AUC or NRMSE (%) | Variation |
| Binary part | *C. bolitinos* | 330.5 | 0.886 | 0.870 | 0.016 | 0.836 | 0,05 |
|  | *C. enderleini* | 154.5 | 0.949 | 0.925 | 0.024 | 0.813 | 0,136 |
|  | *C. grahamii* | 312.1 | 0.884 | 0.850 | 0.034 | 0.743 | 0,141 |
|  | *C. imicola* | 261.7 | 0.929 | 0.914 | 0.015 | 0.847 | 0,082 |
|  | *C. kibatiensis* | 322 | 0.896 | 0.878 | 0.018 | 0.836 | 0,06 |
| Count part | *C. bolitinos* | 1098.2 | 16% | 15% | -1% | 11.8% | -4.2% |
|  | *C. enderleini* | 295.2 | 17% | 28.6% | 11.6% | 53.2% | 36.2% |
|  | *C. grahamii* | 556.8 | 13.4% | 20.7% | 7.3% | 21% | 7.6% |
|  | *C. imicola* | 2280 | 9% | 15.3% | 6.3% | 8.1% | -0.9% |
|  | *C. kibatiensis* | 1888.8 | 11.3% | 13.6% | 2.3% | 14% | 2.7% |
| Final mixed effect NBH model | *C. bolitinos* | 948.2 | 8.5% | 10.4% | 1.9% | 7.6% | -0.9% |
|  | *C. enderleini* | 409.5 | 4.4% | 6.4% | 2% | 17.6% | 13.2% |
|  | *C. grahamii* | 705.4 | 4.9% | 5.9% | 1% | 11.6% | 6.7% |
|  | *C. imicola* | 1222 | 4.8% | 5.1% | 0.3% | 6.1% | 1.3% |
|  | *C. kibatiensis* | 1122.7 | 5.9% | 6.3% | 0.4% | 10.7% | 4.8% |

AUC for binary part, NRMSE for count part and final mixed effect NBH model.
